# Supplementary material for: Use of an oversized AAV8 vector for CPS1 deficiency results in long-term survival and ammonia control
Source: Mol Ther Nucleic Acids. 2025 Feb 3;36(1):102470. doi: 10.1016/j.omtn.2025.102470 (PMC11905892; doi:10.1016/j.omtn.2025.102470)
Supplement: Document S1. Figures S1 and S2 [file mmc1.pdf]

OMTN, Volume *36*

Supplemental information

Use of an oversized AAV8 vector for CPS1 deficiency results in long-term survival and ammonia control

Taryn Diep, Wesley Zhou, Rachel E. Reyes, Matthew Nitzahn, Isabel L. Day, Georgios Makris, Lindsay Lueptow, Irina Zhuravka, Stuti Bakshi, Jon Gangoiti, Hyacinth Padaon, Yunfeng Li, Bruce A. Barshop, Johannes Haberle, and Gerald S. Lipshutz

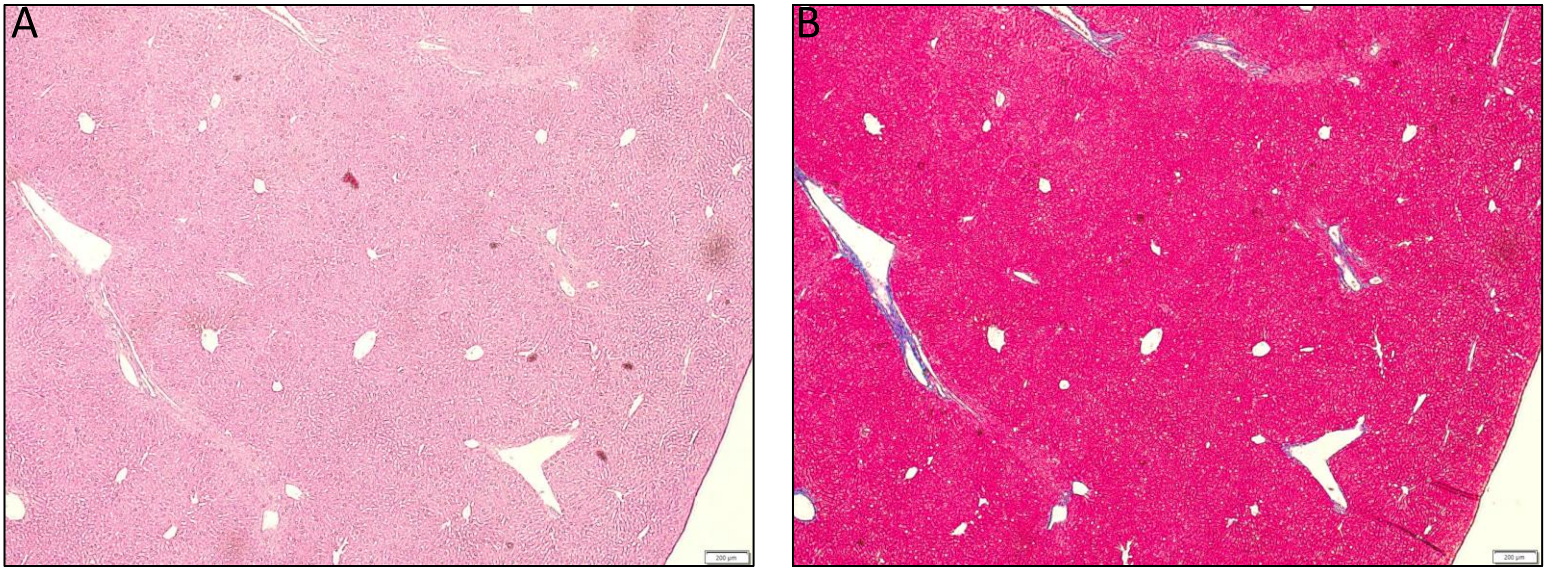

Figure S1. Representative images of age matched Cps1$^{flox/flox}$ liver H&E staining (**A**) demonstrate normal hepatic architecture without lymphocytic infiltrates or evidence of fibrosis on trichrome staining (**B**). Scale bar is 200 μm.

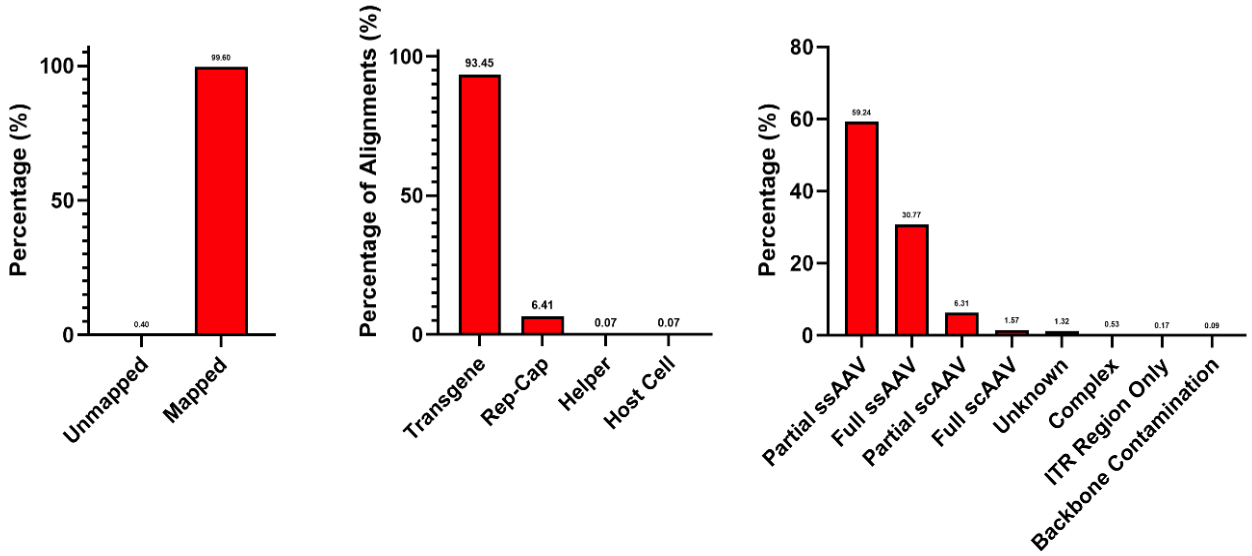

Figure S2. *AAV8.Null Genome Length Fragmentation.* AAV8.Null was barcoded and sequenced using Oxford Nanopore Ligation Sequencing Kit, SQK-LSK114, on a single PromethION™ Flow Cell, and 99.60% of fragments aligned to the created reference sequence (combination of host, helper plasmid, Rep-cap plasmid, and transgene plasmid sequences) **(A)** with a vast majority of alignments (93.45%) mapping to the targeted transgene sequence **(B)**. The percentages of the various AAV transgene genome types indicate the AAV8.Null fragments identified in the sample **(C)**.
