# Supplementary material for: Use of an oversized AAV8 vector for CPS1 deficiency results in long-term survival and ammonia control
Source: Mol Ther Nucleic Acids. 2025 Feb 3;36(1):102470. doi: 10.1016/j.omtn.2025.102470 (PMC11905892; doi:10.1016/j.omtn.2025.102470)
Supplement: Document S2. Article plus supplemental information [file mmc2.pdf]

Molecular Therapy
Nucleic Acids
Original Article

Use of an oversized AAV8 vector for CPS1 deficiency results in long-term survival and ammonia control

Taryn Diep,[1] Wesley Zhou,[1] Rachel E. Reyes,[1,2,3] Matthew Nitzahn,[4] Isabel L. Day,[1,5] Georgios Makris,[6] Lindsay Lueptow,[7] Irina Zhuravka,[7] Stuti Bakshi,[1] Jon Gangoiti,[8] Hyacinth Padaon,[8] Yunfeng Li,[9] Bruce A. Barshop,[8] Johannes Haberle,[6] and Gerald S. Lipshutz[1,4,5,10,11,12]

[1]Department of Surgery, David Geffen School of Medicine at UCLA, Los Angeles, CA, USA; [2]Department of Pathology, Children's Hospital, Los Angeles, CA, USA; [3]School of Pharmacy, University of Southern California, Los Angeles, CA, USA; [4]Molecular Biology Institute, David Geffen School of Medicine at UCLA, Los Angeles, CA, USA; [5]Department of Molecular and Medical Pharmacology, David Geffen School of Medicine at UCLA, Los Angeles, CA, USA; [6]Division of Metabolism and Children's Research Center, University Children's Hospital Zurich, Zurich, Switzerland; [7]Department of Psychology, David Geffen School of Medicine at UCLA, Los Angeles, CA, USA; [8]Department of Pediatrics, Division of Biochemical Genetics, University of California, San Diego, San Diego, CA, USA; [9]Department of Pathology and Laboratory Medicine, David Geffen School of Medicine at UCLA, Los Angeles, CA, USA; [10]Department of Psychiatry, David Geffen School of Medicine at UCLA, Los Angeles, CA, USA; [11]Intellectual and Developmental Disabilities Research Center at UCLA, David Geffen School of Medicine at UCLA, Los Angeles, CA, USA; [12]Department of Semel Institute for Neuroscience, David Geffen School of Medicine at UCLA, Los Angeles, CA, USA

Carbamoyl phosphate synthetase 1 (CPS1) deficiency, a urea-cycle disorder, results in hyperammonemia initiating a sequence of adverse events that can lead to coma and death if not treated rapidly. There is a high unmet need for an effective therapeutic for this disorder, especially in early neonatal patients where mortality is excessive. However, development of an adeno-associated virus (AAV)-based approach is hampered by large cDNA size and high protein requirement. We developed an oversized AAV vector as a gene therapy to treat *CPS1* deficiency. In order to constrain genome size, we utilized small liver-specific promoter/enhancers and a minimal polyadenylation signal. Long-term survival (9 months, end of study) with ammonia control was achieved in AAV8.CPS1-administered Cps1$^{flox/flox}$ mice, while all null vector-injected controls died with marked hyperammonemia; female mice demonstrated improved survival over treated males. While glutamine remained elevated compared to controls, ammonia was controlled in surviving animals. Mice maintained their weights and were not sarcopenic. While drinking water did contain carglumic acid, no nitrogen scavengers were administered. Although there were concerns with vector genomic integrity, these findings demonstrate proof of concept for an oversized gene-therapy approach for a challenging urea-cycle disorder where high-level hepatic protein is essential for survival.

INTRODUCTION

The urea cycle is the biochemical pathway for disposal of excess nitrogen into non-toxic urea in terrestrial mammals. It consists of six enzymes and two transporter proteins; mutations in any of these can result in a urea-cycle disorder (UCD).[1] Carbamoyl phosphate synthetase 1 (CPS1; E.C. 6.3.4.16) catalyzes the first step in the cycle, one that is irreversible and rate limiting under normal physiologic conditions; it is an ATP-dependent conversion of ammonia, glutamine, and bicarbonate into carbamoyl phosphate and ADP in a three-step reaction.[2–4] This synthesis occurs in the mitochondria of hepatocytes and is dependent on the allosteric activator N-acetylglutamate (NAG), and Mg^{2+}.[5] CPS1 itself is a highly abundant enzyme representing ∼20% of the mitochondrial protein and ∼5% of total liver protein.[6] As an autosomal recessive deficiency (OMIM: 237300), it leads to reduced or absent CPS1 enzymatic activity, the consequences of which are usually acutely devastating to the brain (progressing from disorientation and somnolence to combativeness, seizures, and coma),[7] and has the potential for early neonatal death within the initial hyperammonemic episode. Intellectual disability is common in survivors.[8] In general, neonatal hyperammonemic coma due to a UCD has a mortality of 46% with 79% of survivors having developmental disability.[9] While estimates of the incidence vary, CPS1 deficiency is thought to occur with a rate of 1 in 1.3 million births in the United States and Europe; however, it is proposed that this is an underestimate due to challenges in making an early diagnosis.[10]

Present-day therapy is of two forms. First, with an acute and life-threatening metabolic decompensation episode, hemodialysis may be necessary to aggressively reduce plasma ammonia and prevent neonatal death. Beyond the acute period, the goal is to prevent recurrent episodes through chronic treatment strategies, including a

Received 8 August 2024; accepted 28 January 2025;
https://doi.org/10.1016/j.omtn.2025.102470.

Correspondence: Gerald S. Lipshutz, Department of Surgery, David Geffen School of Medicine at UCLA, 757 Westwood Plaza, Room 8501G, Los Angeles, CA 90095-7054 USA.
E-mail: glipshutz@mednet.ucla.edu

Published by Elsevier Inc. on behalf of The American Society of Gene and Cell Therapy.
This is an open access article under the CC BY license (http://creativecommons.org/licenses/by/4.0/).

protein-restricted diet, sufficient calories to prevent catabolism,[11] nitrogen-scavenger administration, and supplementation with L-arginine and L-citrulline.[12] Afflicted patients with residual CPS1 enzymatic activity may benefit from prolonged oral administration of carglumic acid, the deacylase-resistant analog of NAG, as activator-saturation therapy.[13] However, even in the best of treatment circumstances, afflicted patients remain vulnerable to nitrogen. Prevention is essential to overall neurological outcomes because brain injury that occurs from acute hyperammonemic crises is irreversible. Presently, the only truly effective treatment for this single-gene disorder is liver transplantation[14–16] with its incumbent long-term risks of infection, malignancy, atherosclerotic disease, and renal insufficiency primarily due to chronic pharmacologic immunosuppression.

There are several challenges to developing a gene-based approach for CPS1 deficiency. First, until relatively recently, there was no animal model available for investigation. A neonatal murine model was developed, but afflicted mice died within 24 h of birth due to hyperammonemia likely due to maternal milk intake.[17] An alternative model was established to overcome this challenge, in which adult mice recapitulate the disease phenotype after Cre-mediated deletion of *Cps1*.[18] While latent in its onset, this model has proved vital to exploring new approaches and improving our understanding of the disorder.[18,19] Second, therapeutic enzyme levels are relatively high.[1] CPS1 is an abundant mitochondrial matrix protein expressed ubiquitously throughout the liver. Therefore, achieving an ample amount of *CPS1* expression is necessary for an effective gene-therapy approach. Finally, and most formidable, is consideration of the large size of the *CPS1* cDNA at 4.5 kb. It is challenging to develop an adeno-associated virus (AAV) vector, with a wild-type viral genome size of 4.7 kb,[20] that is able to produce a clinically translatable hepatotropic virus containing a *CPS1* expression cassette. There are numerous examples of recombinant AAVs being successfully developed for study, both in preclinical models and as approved human therapeutics[21–23]; however, the limited packaging capacity has been an impediment for certain disorders where the cDNA is substantially large. While this obstacle has been overcome at some level for certain genetic disorders, such as Duchenne muscular dystrophy,[24] cystic fibrosis,[25] and hemophilia A,[26] by removal of specified genetic sequences where the coding sequence length is about equal or would exceed the wild-type AAV genome size, this strategy cannot be applied successfully for CPS1 as the complex enzyme function is dependent on its intact native sequence. Thus, this requirement permits little space for regulatory components in the expression cassette.

The possibility of packaging a limited oversized expression cassette genome into an AAV8 capsid combined with the ability to efficiently transduce the liver could allow for the development of a method to treat CPS1 deficiency while avoiding the inefficiency of a dual-vector, homologous-recombination-dependent approach.[19] We describe herein the implementation of a single-vector approach in developing an AAV vector to treat CPS1 deficiency. We developed a vector with a small hepatocyte-specific promoter enhanced by a minimal transcriptionally active *cis*-regulatory module. We also employed a small synthetic 5′ intron to mediate enhancement of expression while having a minimal synthetic polyadenylation signal to promote translation and maintain mRNA stability. We demonstrate that this AAV approach, while limited in its efficacy and with vector production challenges, can restore hepatic CPS1 expression, maintain stable plasma ammonia control, and promote ureagenesis. Treated *Cps1*$^{flox/flox}$ mice are viable long term and reduce their plasma ammonia after undergoing an exogeneous ammonium challenge; however, they do remain nitrogen vulnerable. These studies are the first step in establishing the groundwork and proof of principle for a potential clinically translatable single-vector AAV gene-therapy approach for CPS1 deficiency.

RESULTS

Administration of AAV8.CPS1, unlike AAV8.Null, leads to long-term survival in Cps1 deficiency

A serotype 8 AAV (AAV8) was developed to express human codon-optimized *CPS1* (AAV8.CPS1) in hepatocytes (Figure 1A). As the cDNA of *CPS1* (4.5 kb) is large with a sequence that is not modifiable other than codon optimization, all other genetic components were kept as small as possible to control overall AAV genome size. The other *cis*-acting components include the HSCRM8 enhancer (71 bp), the transthyretin (TTR) promoter (235 bp), a synthetic 5′ intron (108 bp), and a synthetic polyadenylation signal (46 bp). With the left and right inverted terminal repeats (ITRs) from AAV2 (141 bp each) the obligate genome size was 5263 bp.

Adult *Cps1*$^{flox/flox}$ mice[18] (2 months old) were intravenously administered AAV8 expressing Cre recombinase with either (1) AAV8 expressing human codon-optimized *CPS1* (male, n = 6, female n = 8) or (2) AAV8.Null (male, n = 5, female n = 5) (day 0) (Figure 1B) that lacks a transgene. *Cps1*$^{flox/flox}$ mice without AAV8.Cre recombinase administration were injected with vehicle and served as Cps1 wild-type controls (male, n = 5; female n = 5). Plasma was collected before injection and on days 10, 21, and 30 and monthly thereafter in surviving mice for the length of study (9 months). Carglumic acid (0.1%) with dextrose (5%) was added to drinking water beginning 14 days after AAV was administered. Eight months after administration, behavioral phenotyping was performed in all mice, while at 9 months mice underwent ureagenesis determination and ammonia challenge followed by euthanasia and tissue analysis.

To characterize health and survival endpoints, mice were weighed (weight being a surrogate of health) and examined daily after vector administration. All female and male untreated Cps1 wild-type control mice survived to 9 months (end of study) (Figures 2A and 2B). *Cps1*$^{flox/flox}$ mice that received AAV8.Null with AAV8.cre recombinase died; all male mice died by day 27 (Figure 2A), while all female mice died by day 36 (Figure 2B). However, more than 50% of the male mice survived to nearly 6 months and seven of eight female mice survived to 9 months after vector administration ($p < 0.0001$ across both sexes). While AAV8.Null with AAV8.cre recombinase mice developed progressive weight loss and sarcopenia beginning at 14 days after administration of vectors (Figures 2C and 2D, red squares), AAV8.CPS1 mice demonstrated stability of weight throughout the

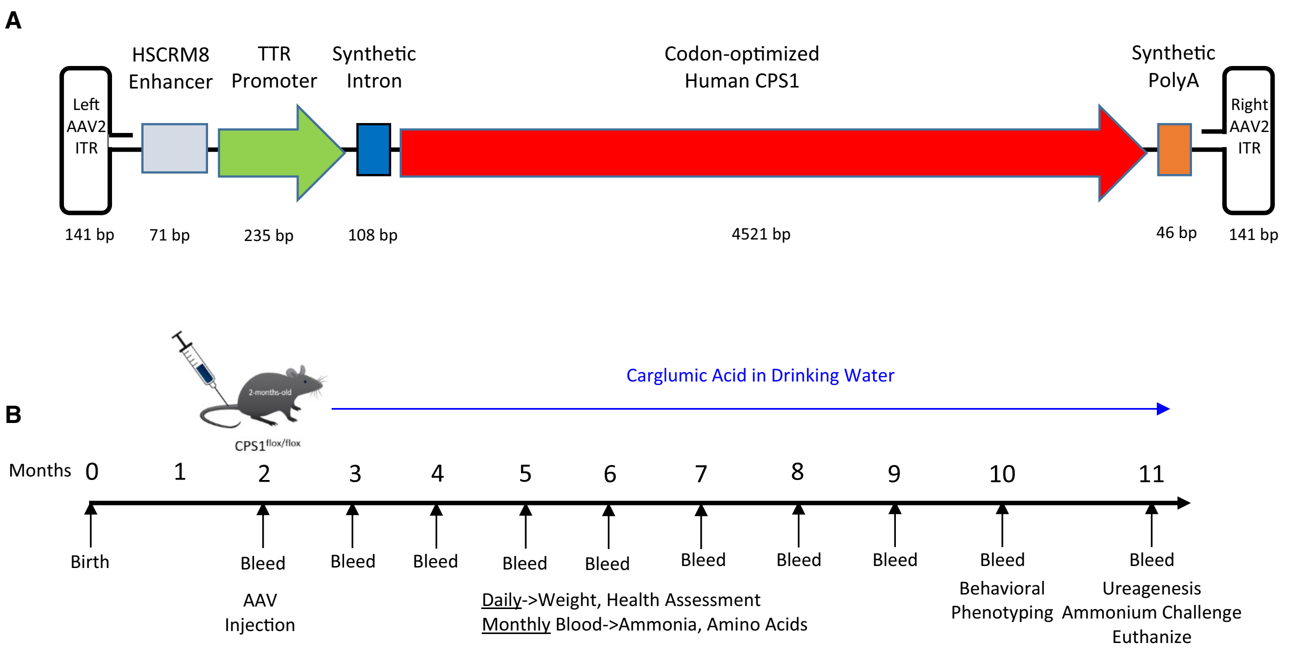

Figure 1. Development of an oversized AAV for CPS1 expression and outline of experiments

(A) A serotype 8 adeno-associated viral vector was developed to express human codon-optimized *CPS1* in hepatocytes. Due to the large cDNA size of *CPS1* (4.5 kb), all other genetic components were kept as small as possible to control overall AAV genome size: HSCRM8 enhancer (71 bp), transthyretin (TTR) promoter (235 bp), a synthetic 5′ intron (108 bp), and a synthetic polyadenylation signal (46 bp). With the left and right inverted terminal repeats from AAV2 (ITR) (141 bp each), the obligate genome size was 5263 bp. (B) Adult *Cps1*$^{flox/flox}$ mice (2 months old) were intravenously administered AAV8 expressing Cre recombinase with or without AAV8 expressing human codon-optimized *CPS1* and analyzed each 30 days after injection. Carglumic acid (0.1%) in water with dextrose (5%) was made available in drinking water. Eight months after administration, behavioral phenotyping was performed, and, at 9 months, mice underwent ureagenesis determination and ammonia challenging followed by euthanasia and tissue studies. HSCRM, hepatocyte-specific transcriptional *cis*-regulatory module; CPS1, carbamoyl phosphate synthetase 1; TTR, transthyretin; PolyA, polyadenylation signal; ITR, inverted terminal repeat.

studies (blue triangles). Mice were physically compared for size and weight at 9 months. Male AAV8.CPS1-treated mice appear smaller (Figure 2E, picture) and demonstrated reduced weights compared to age-matched controls (at 11 months, 26.62 ± 0.11 g vs. 35.17 ± 2.21 g, respectively; $p = 0.0009$) (Figure 2E, graph); female AAV8.CPS1-treated mice, however, appeared of similar size (Figure 2F, picture) and were not statistically different in weight compared to age-matched controls (25.29 ± 0.83 g vs. 30.91 ± 5.41 g; $p = 0.08$) (Figure 2F, graph).

AAV8.CPS1 normalizes alanine aminotransferase and prevents hyperammonemic coma in Cps1-deficient mice

Examination for liver pathology was conducted in vector-administered mice and controls. Representative hematoxylin & eosin (H&E) staining (Figure 3A) showed no evidence of necrosis or infiltrates; trichrome staining (Figure 3B) demonstrated no evidence of fibrosis or abnormal collagen deposition. *Cps1*$^{flox/flox}$ livers from control mice lacked any evidence of liver pathology. Representative images are presented in Figure S1.

In situ hybridization (Figure 3C [red pigment]) demonstrated widely distributed mRNA of the *CPS1* transgene in transduced hepatocytes from experimental mice. However, immunohistochemical detection of vector-derived CPS1 in AAV-transduced murine liver demonstrated primarily transgene-encoded protein being present in perivenous hepatocytes; expression was also detected in scattered interlobular hepatocytes, albeit substantially less than perivenous expression (Figure 3D). Human CPS1 protein was detected by western blot. In order to obtain images where bands could be visualized for comparison and using the same imaging settings, anti-CPS1 antibody concentrations were optimized for each mouse group (see section materials and methods). Vector-derived CPS1 protein is substantially less than wild type (a representative western blot image is presented in Figure 3E). To determine the amount of vector-derived CPS1 in the liver of AAV8.CPS1-injected mice, we performed a human-specific CPS1 ELISA. Human CPS1 detected in the AAV8.CPS1-treated livers 9 months after vector administration was 0.368 ± 0.128 μg CPS1/mg total protein (n = 10 livers [both sexes represented], performed in duplicate).

Plasma was collected from vehicle-only controls, AAV8.Null with AAV8.Cre, and AAV8.CPS1 with AAV8.Cre mice. Alanine aminotransferase (ALT) values were markedly elevated in mice with untreated Cps1 deficiency (217.3 ± 64.8 units/L, $n = 8$ [red squares])

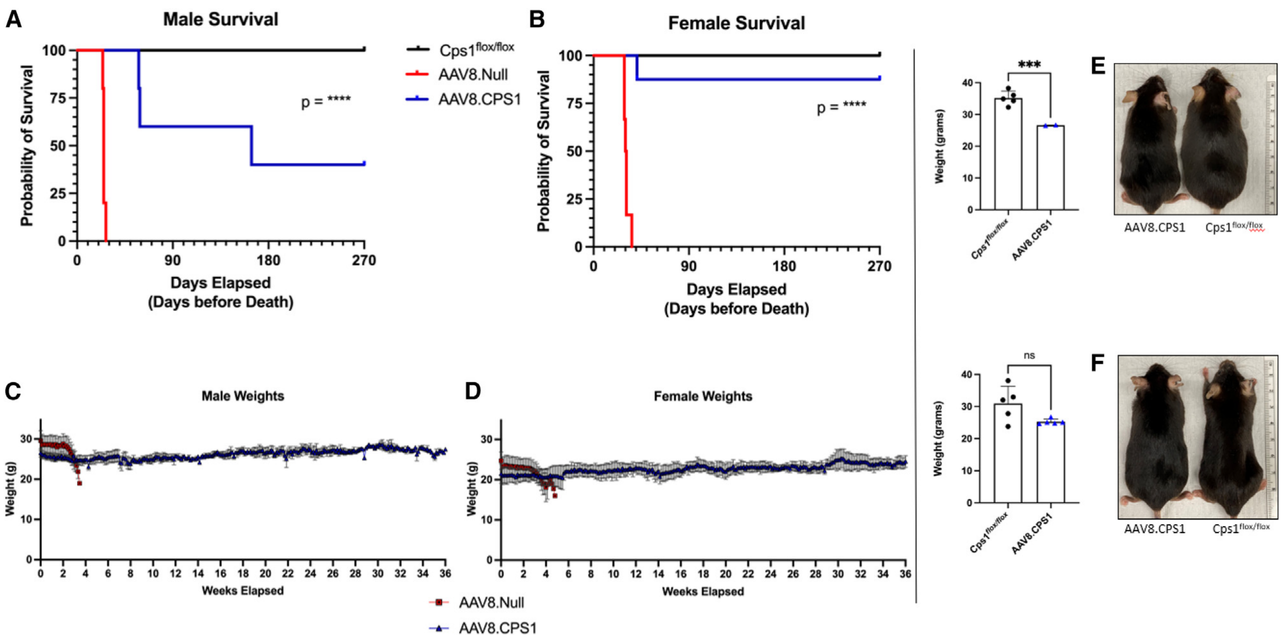

Figure 2. AAV-CPS1 administration results in long-term mouse survival and promotes stable weights
Adult *Cps1*$^{flox/flox}$ mice were intravenously administered vehicle only (black), vehicle with AAV8 expressing Cre recombinase and AAV8 with no transgene (i.e., null) (red), or vehicle with AAV8 expressing Cre recombinase and AAV8 expressing human codon-optimized *CPS1* (blue) and followed daily for 9 months. Survival was followed in both (A) male and (B) female mice. Mice were weighed daily (C and D) to examine for evidence of sarcopenia and as a surrogate for health. Before euthanasia at 11 months of age, weights from surviving male (E) and female (F) mice (blue triangles) were obtained and compared with vehicle-only-administered controls (black circles). Data are presented as are mean ± SD. ***p = 0.0001–0.001; ****p < 0.0001; ns, not significant

compared to vehicle-only injected controls (39.40 ± 21.4 U/L n = 10 [black circles]) (p < 0.0001). However, plasma ALT values from AAV CPS1-treated mice (37.1 ± 14.9 U/L, n = 7 [blue triangles]) were not statistically different from vehicle-only-administered controls (p = 0.8014) (Figure 3F).

AAV8.CPS1 establishes ureagenesis and a successful response to ammonium challenging

Isotopic methods were utilized to determine the ability of AAV-mediated CPS1 hepatic expression to restore ureagenesis. Using ^{13}C-acetate administration to determine the ureagenesis rate in both Cps1 wild-type control and AAV-treated mice at 9 months after vector administration, male mice achieved ureagenesis of 1.78 ± 2.33 μM/h (n = 3) compared to control mice with 18.39 ± 5.83 μM/h (n = 5) (or 9.7% of controls) (Figure 3G); female AAV-treated mice achieved 1.18 ± 0.40 μM/h (n = 8) compared to control mice with 14.97 ± 3.37 μM/h (n = 5) (or 7.9% of controls) (Figure 3H). Plasma urea was measured at intervals throughout the study (Figure 3I). In AAV-treated mice, plasma urea was 19.5% of controls (1,501.15 ± 456.09 μmol/L compared to 7,690.01 ± 1,264.62 μmol/L; p < 0.0001) in Cps1 wild-type mice.

Plasma ammonia (Figure 3J) was stable in the Cps1 wild-type control mice (black circles) throughout the 9 months of study, while the AAV8.Null mice, not unexpectedly, had a marked rise after day 10 (red squares); data collection ceased as all mice expired or were euthanized for a humane endpoint. AAV8.CPS1-treated mice, however, were generally stable (blue triangles) (except in male mice around day 60 where 2 had elevated plasma ammonia and died shortly thereafter without evidence of weight loss or sarcopenia). At 9 months after administration, plasma ammonia was stably maintained in AAV8.CPS1-treated mice at ∼2 times that of Cps1 wild-type control mice (455.65 ± 100.06 μmol/L vs. 231.97 ± 28.09 μmol/L; p = 0.0008).

In all surviving mice 9 months after vector or vehicle administration, an ammonium challenge was performed to examine for development of abnormal neurological events and evidence of a therapeutic ammonium metabolic response from acute loading. Fifteen minutes after ammonia loading by intraperitoneal administration, behavioral testing (scale 1–7) revealed no statistically significant difference in behavior scores (Figure 3K): AAV8.CPS1-treated mice in general did not demonstrate evidence of spontaneous or sound-induced seizures, hyperacute response to auditory stimuli, or altered gait (score 6.50 ± 0.53 [AAV treated, n = 10]) compared to Cps1 wild-type control mice (6.70 ± 0.48 [Cps1 wild-type control, n = 10]; p = 0.39). Blood ammonia levels were determined in mice immediately before ammonium chloride administration and at 20 and 60 min post administration (Figure 3L). Not unexpectedly, baseline blood ammonia levels were elevated in AAV8.CPS1-treated mice: 232.80 ± 111.75 μmol/L (n = 11) compared to Cps1 wild-type

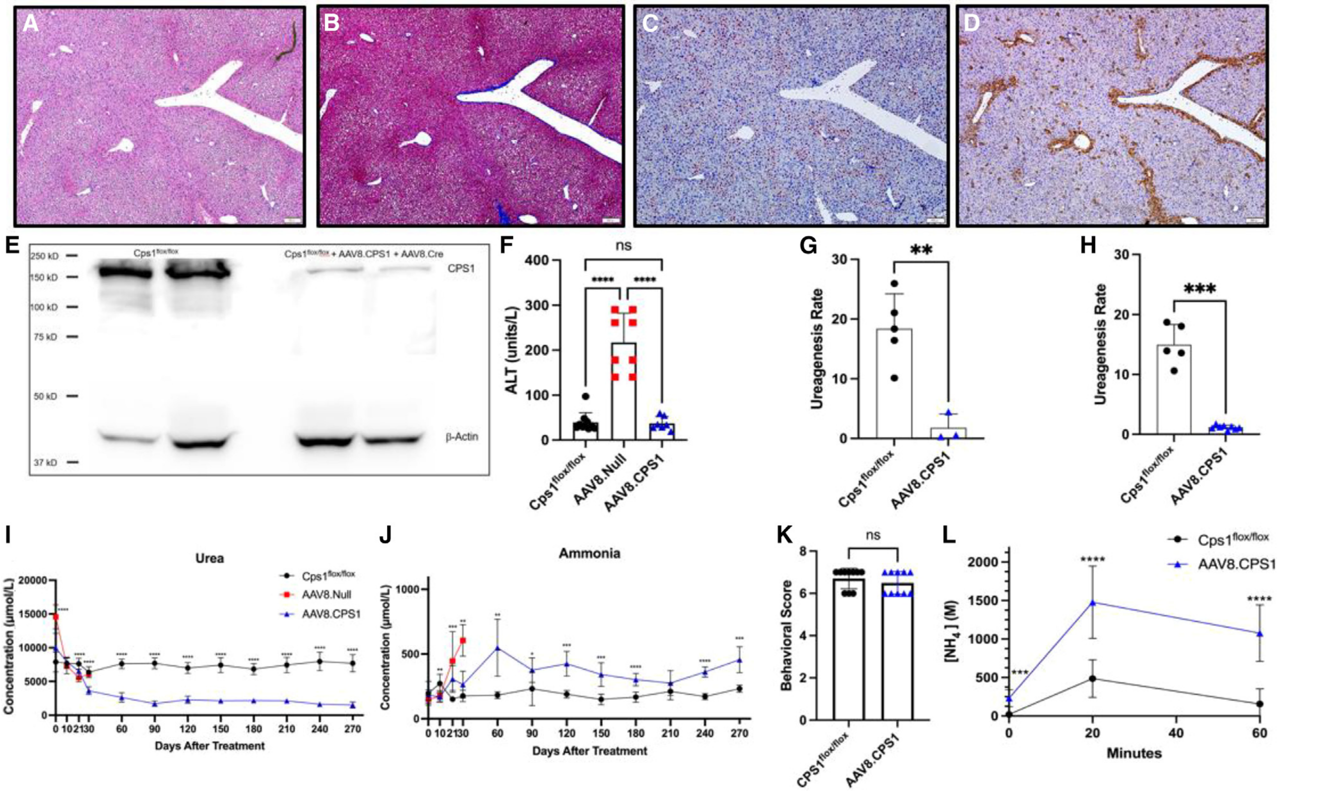

Figure 3. AAV.CPS1 administration results in absence of liver pathology, normalizes plasma ALT levels, and results in long-term ureagenesis and ammonia stability

Nine months after AAV administration (11 months of age), plasma and liver were collected for analysis. Representative images of liver H&E staining (A) demonstrate normal hepatic architecture without lymphocytic infiltrates or evidence of fibrosis on trichrome staining (B). A representative image of *in situ* hybridization (C) specific for human codon-optimized RNA shows expression in human liver as represented by the red precipitates. CPS1 protein expression is most prominent in hepatocytes near the hepatic veins in AAV-treated Cps1 mice as represented by the brown precipitate (D). Western blotting (E) demonstrates evidence of hepatic expression in AAV-treated CPS1 mice (see section materials and methods for details of blot). Measurement of plasma alanine aminotransferase (ALT) demonstrated elevation in AAV-Cre recombinase-only-treated mice (red circles) (F) (217.3 ± 64.8 [mean +SD], male [$n = 4$] female [$n = 4$]) compared to vehicle-only controls (black circles) (39.40 ± 21.4 U/L [mean +SD], male [$n = 5$], female value [$n = 5$], $p < 0.0001$) and AAV8.CPS1-administered and surviving mice (blue circles) (37.1 ± 14.9 [mean +SD], male [$n = 2$], female [$n = 5$]). There was no statistical difference between AAV-CPS1-treated mice and vehicle controls ($p = 0.8014$). Ureagenesis, measured with ^{13}C-acetate, while present, was reduced in both males (G) at 9.7% of controls and females (H) at 7.9% of the control rate. Plasma urea, as the principal nitrogenous waste product of metabolism generated through the urea cycle, while reduced, is produced throughout the study (I). Plasma ammonia (J) is elevated but demonstrates control (compared to vehicle-only injected [black] mice) in AAV8.CPS1-treated (blue) mice compared to mice only receiving AAV8.Cre and vehicle (red) where all died due to hyperammonemia. After administration of ammonium chloride, limited behavioral testing at 20 min demonstrates no differences between groups (K). Plasma ammonia levels (L) in AAV8.CPS1 mice (in blue) begin to decrease over 60 min in mice, although this occurs less rapidly in AAV8.CPS1-treated mice when compared to vehicle-only controls (in black). Data are presented as mean ± SD. *$p \leq 0.05$, **$p \leq 0.01$, ***$p = 0.0001$–0.001, and ****$p < 0.0001$. ALT, alanine aminotransferase; NS, not significant; Cre, cre recombinase; CPS1, carbamoyl phosphate synthetase 1. (A–D) Scale bar, 200 μm.

controls: 21.50 ± 11.26 μmol/L ($n = 10$) ($p < 0.0002$). At 20 min after ammonium administration, there was a marked increase in blood ammonia levels in AAV8.CPS1-treated mice: 1,478.50 ± 468.70 μmol/L vs. 487.30 ± 243.94 μmol/L in controls ($p < 0.0001$). At 60 min, AAV-treated mice did show a metabolic response with a decline in blood ammonia levels by 27% (to 1,076.80 ± 336.05 μmol/L); not unexpectedly, Cps1 wild-type control mice showed a more pronounced metabolic response with a decline of 68% (to 156.50 ± 196.64 μmol/L; $p < 0.0001$ comparing groups).

Long-term modest control of urea-cycle-related amino acids is achieved with AAV8.CPS1 administration

Plasma amino acids were measured at baseline; days 10, 21, and 30; and monthly thereafter to 9 months (Figures 4A–4H). Urea-cycle-related amino acids (Figures 4A–4G) and lysine (Figure 4H) were generally stable and paralleled Cps1 wild-type controls. Glutamine, alanine, and asparagine, acting as ammonia buffers, were elevated (at 9 months after AAV8.CPS1 administration, glutamine, 1,556.44 ± 239.46 μmol/L vs. 875.42 ± 198.18 μmol/L, $p = 0.0001$; alanine, 1,024.25 ± 589.32 μmol/L vs. 533.83 ± 195.85 μmol/L, $p = 0.082$; asparagine,

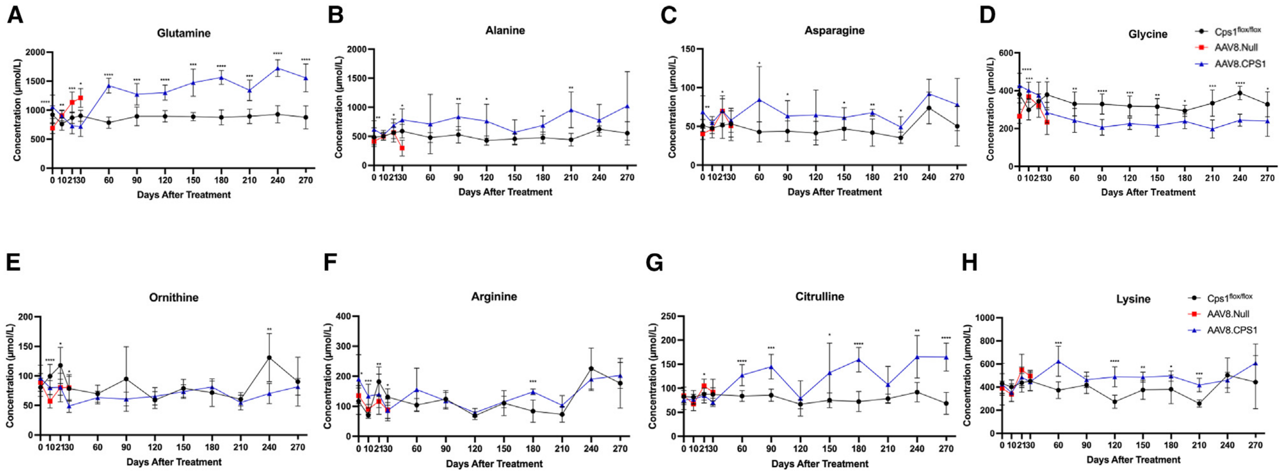

Figure 4. AAV-CPS1 treatment controls some urea-cycle-related amino acid imbalances in mice with CPS1 deficiency
Note sustained elevation of glutamine (A), while alanine (B) and asparagine (C), as other reservoirs of waste nitrogen, are only mildly elevated and glycine is reduced (D). The urea-cycle amino acids ornithine (E) and arginine (F) are not substantially different between vehicle-only controls and AAV8.CPS1-treated mice. Plasma citrulline was moderately elevated (G) in mice as was lysine (H). Data are presented as mean + SD. AAV, adeno-associated virus; Cre, Cre recombinase; CPS1, carbamoyl phosphate synthetase 1. Data are presented as mean ± SD. *$p \leq 0.05$, **$p \leq 0.01$, ***p = 0.0001–0.001, and ****$p < 0.0001$.

78.13 ± 33.86 µmol/L vs. 50.36 ± 25.66 µmol/L, p = 0.095; all AAV8.CPS1 vs. Cps1 wild-type controls, respectively). Glycine was reduced (AAV8.CPS1 239.78 ± 80.95 vs. Cps1 wild-type controls 328.42 ± 65.53; p = 0.035) and serine was normal (AAV8.CPS1, 200.34 ± 51.00 vs. Cps1 wild-type controls, 181.75 ± 64.47; p = 0.518).

Regarding the urea-cycle amino acids ornithine, arginine, and citrulline in AAV8.CPS1-treated mice, the levels are more similar to Cps1 wild type in both sexes at 9 months after AAV8.CPS1 administration (ornithine, 81.53 ± 14.04 µmol/L vs. 90.21 ± 41.43 µmol/L, p = 0.55 [90% on average]; arginine, 202.81 ± 43.27 µmol/L vs. 176.72 ± 82.77 µmol/L, p = 0.41 [115% on average]; citrulline, 164.85 ± 29.11 µmol/L vs. 68.60 ± 22.74 µmol/L, $p < 0.0001$ [240% on average]; all AAV8.CPS1 vs. Cps1 wild-type controls, respectively). Absolute values of lysine were also similar between treatment groups (at 9 months after AAV8.CPS1 administration, 608.24 ± 164.67 µmol/L vs. 442.65 ± 230.33 µmol/L, p = 0.10 [137% on average]; all AAV8.CPS1 vs. Cps1 wild-type controls, respectively).

Behavioral phenotyping demonstrates presence of anxiety and some altered memory acquisition in long-term gene-therapy-treated CPS1-deficient mice

Behavioral phenotyping was performed 8 months after vector administration (n = 11, AAV8.CPS1 treated; n = 10, vehicle-only Cps1 wild-type controls). The SmithKline Beecham, Harwell, Imperial College, Royal London Hospital, phenotype assessment (SHIRPA) primary screen testing physical appearance, locomotion, and sensory reflex reactions was performed to characterize the general phenotype of AAV8.CPS1-treated mice and compare with Cps1 wild-type controls. Measures of activity levels, autonomic responses and reflexes, gait and locomotion, balance, coordination, muscle tone, strength, fear, and aggression were all quantitatively assessed. Of these 33 measures, there were only two statistically different findings in the male mice (reduced startle response, increased lacrimation) and four in the female mice (reduced spontaneous activity, increased trunk curl, and increased response to whisker brush and wire maneuver) (Table 1).

Mice were subjected to spontaneous novel-object recognition (NOR) testing to assess recognition memory (Figure 5A). As mice have an innate predisposition for novelty,[27] they have a propensity to explore a new object rather than a familiar one, which can be quantified using the discrimination index. The discrimination index is defined as the time spent exploring the new object minus the time exploring the familiar object, divided by total exploration time ($[T_{(new)} - T_{(old)}]/T_{(total)}$). Male and female AAV8.CPS1-treated mice demonstrated similar discrimination indices compared to wild-type mice (males, 0.203 ± 0.275 vs. 0.225 ± 0.113, controls [n = 8] vs. AAV8.CPS1-treated [n = 3], respectively [p = 0.7561]; females, 0.219 ± 0.279 vs. 0.233 ± 0.207, controls [n = 7] vs. AAV8.CPS1-treated [n = 8] [p = 0.9087]). These data indicate that recognition learning and memory in treated Cps1-deficient mice are without deficit as compared to Cps1 wild-type mice.

Contextual fear conditioning provides an assessment of associative learning between a stimulus (i.e., foot shock) and the environmental context. The dependent measure is a freezing response. For acquisition, mice are placed in a chamber and shocked three times. The next day, they are returned to the same environment and the freezing response is measured. Due to group sizes, sexes were combined for analysis (Figures 5B–5D). There were no differences in either baseline freezing or acquisition between the AAV8.CPS1-treated group and controls (Figure 5B). However, during the context test, there was decreased total freezing (Figure 5C) in the AAV8.CPS1-treated group (73.48% ± 12.12% vs. 52.51% ± 23.69% [n = 16 controls, n = 11

Table 1. SHIRPA testing

	Cps1$^{flox/flox}$ (M)	AAV.CPS1 (M)	*p* value	*Cps1*$^{flox/flox}$ (F)	AAV.CPS1 (F)	*p* value
Viewing jar						
Body position	4.13 ± 0.13	4.00 ± 0.00	0.91	4.0 ± 0.00	3.88 ± 0.13	0.80
Spontaneous activity	1.63 ± 0.26	1.33 ± 0.33	0.91	2.50 ± 0.27	1.13 ± 0.13	0.001
Tremor	0.13 ± 0.13	0.00 ± 0.00	0.78	0.00 ± 0.00	0.00 ± 0.00	>0.99
Urination	0.38 ± 0.18	0.00 ± 0.00	0.48	0.00 ± 0.00	0.250 ± 1.64	0.57
Defecation	2.25 ± 0.73	0.00 ± 0.00	0.51	2.63 ± 1.21	0.88 ± 0.5806	0.47
Arena						
Transfer arousal	5.00 ± 0.00	4.67 ± 0.33	0.26	5.00 ± 0.00	5.13 ± 0.13	0.77
Locomotor activity	31.75 ± 1.52	27.67 ± 2.73	0.65	32.38 ± 1.99	33.63 ± 1.96	0.96
Piloerection	0.123 ± 0.123	0.00 ± 0.00	0.78	0.00 ± 0.00	0.0 ± 0.00	>0.99
Gait	0.0 ± 0.00	0.00 ± 0.00	>0.99	0.13 ± 0.13	0.13 ± 0.13	>0.99
Pelvic elevation	2.25 ± 0.16	2.00 ± 0.00	0.99	2.33 ± 0.33	2.13 ± 0.13	0.90
Tail elevation	1.25 ± 0.16	1.67 ± 0.33	0.35	1.13 ± 0.13	1.0 ± 0.00	0.90
Startle response	0.88 ± 0.13	0.00 ± 0.00	0.02	0.75 ± 0.16	0.25 ± 0.16	0.10
Touch escape	2.75 ± 0.16	2.33 ± 0.33	0.69	2.25 ± 0.16	2.25 ± 0.25	>0.99
Held by tail						
Trunk curl	0.75 ± 0.164	1.00 ± 0.00	0.74	0.25 ± 0.16	1.00 ± 0.00	0.00
Visual placing	2.88 ± 0.13	3.00 ± 0.00	0.99	2.88 ± 0.23	3.00 ± 0.27	0.97
Whisker brush	2.75 ± 0.16	3.00 ± 0.00	0.95	1.75 ± 0.41	2.88 ± 0.13	0.02
Whisker placement	2.27 ± 0.18	3.00 ± 0.00	0.68	2.38 ± 0.18	2.27 ± 0.18	0.74
Reflex						
Grip strength	2.88 ± 0.23	2.00 ± 0.58	0.23	2.75 ± 0.16	2.25 ± 0.25	0.44
Pinna reflex	0.86 ± 0.14	1.00 ± 0.00	0.72	1.00 ± 0.00	1.00 ± 0.00	>0.99
Corneal reflex	0.88 ± 0.13	1.00 ± 0.00	0.78	1.00 ± 0.00	1.00 ± 0.00	>0.99
Toe pinch	2.88 ± 0.23	2.67 ± 0.88	0.99	2.88 ± 0.23	2.27 ± 0.42	0.95
Wire maneuver	2.63 ± 0.26	1.33 ± 1.33	0.47	0.86 ± 0.44	2.75 ± 0.49	0.04
Supine restraint						
Body length	9.69 ± 0.27	9.50 ± 0.29	0.95	9.81 ± 0.13	9.13 ± 0.13	0.07
Heart rate	1.00 ± 0.00	1.00 ± 0.00	>0.99	0.88 ± 0.13	1.00 ± 0.00	0.58
Limb tone	2.25 ± 0.16	2.33 ± 0.33	1.00	1.63 ± 0.26	1.13 ± 0.13	0.29
Lacrimation	0.00 ± 0.00	0.33 ± 0.33	0.04	0.00 ± 0.00	0.00 ± 0.00	>0.99
Provoked biting	1.00 ± 0.00	1.00 ± 0.00	>0.99	0.88 ± 0.13	1.00 ± 0.00	0.58
Penlight vision	0.75 ± 0.16	0.67 ± 0.33	0.99	0.38 ± 0.18	0.88 ± 0.13	0.16
Other						
Righting reflex	0.12 ± 0.13	0.00 ± 0.00	0.95	0.38 ± 0.18	0.00 ± 0.00	0.16
Negative geotaxis	1.38 ± 0.32	2.67 ± 0.67	0.28	1.00 ± 0.38	1.00 ± 0.38	>0.99
Irritability	0.50 ± 0.19	0.33 ± 0.33	0.96	0.50 ± 0.19	0.25 ± 0.16	0.77
Aggression	1.0 ± 0.00	1.00 ± 0.00	>0.99	0.88 ± 0.13	1.00 ± 0.00	0.58
Vocalization	0.38 ± 0.18	0.33 ± 0.33	1.00	0.63 ± 0.18	0.25 ± 0.16	0.47

Results of SHIRPA testing of Cps1 wild-type and AAV8.CPS1-treated mice. Data are presented as mean ± SD.

AAV8.CPS1 treated, respectively]; $p = 0.0056$), and decreased freezing across the whole session (Figure 5D) between Cps1 wild-type and AAV8.CPS1-treated mice. These data suggest that AAV-treated mice have decreased associative memory capabilities.

The open-field testing (Figures 5E–5H), a quantitative measurement of overall locomotor behavior, also measures mouse anxiety by assessing travel in the center of a field versus the periphery during the first 5 min of the test while the field is still a novel environment. The

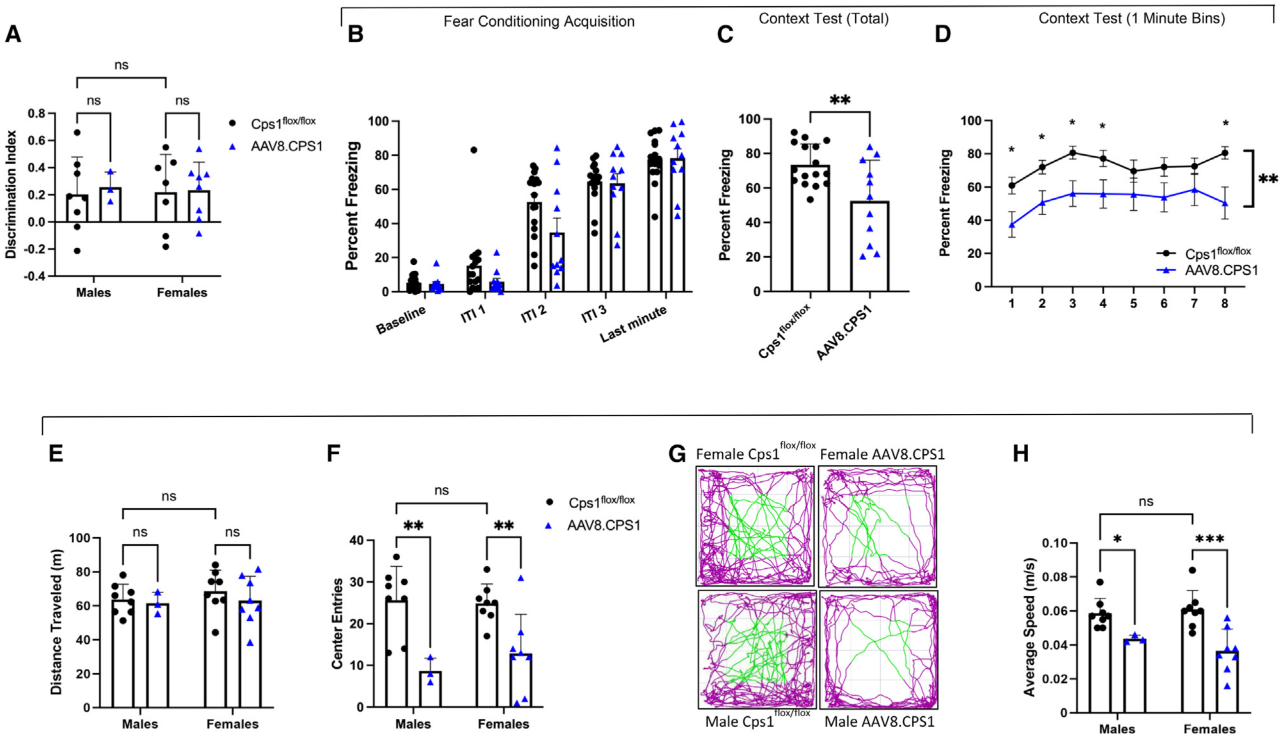

Figure 5. Behavioral testing in AAV-CPS1-treated mice reveals some learning deficits and an anxiety phenotype

Behavioral phenotype testing was performed at 10 months of age (*n* = 11 in AAV8.CPS1-treated group and n = 10 in vehicle-only control group). In NOR (A), there was an absence of statistically significant differences between vehicle-only control (*Cps1*$^{flox/flox}$) (black circles) and AAV8.CPS1-treated mice (blue circles). In fear-conditioning studies of contextual memory where sexes were combined for analysis (B–D), there was decreased freezing overall in the AAV8.CPS1-treated group when mice were re-exposed to the context in which they received shocks. In (B), there were no differences in baseline freezing prior to shocks and freezing during the acquisition (i.e., shock) intertrial intervals (ITIs), demonstrating mice were freezing following shocks. However, upon re-exposure to context 24 h later, total freezing (C) and freezing across the whole session (D) were reduced in the AAV gene-therapy-treated group compared to the Cps1 wild-type controls. In open-field testing (E–H), quantitative measurement of distance traveled (E) was similar between vehicle-only control (*Cps1*$^{flox/flox}$) (black circles) and AAV8.CPS1-treated mice (blue circles). Measurement of entry to the center (F) was reduced with AAV8.CPS1 mice spending more time in the periphery (G) (green line indicates when mice were in the center zone). Average speed of AAV8.CPS1 mice was reduced (H). Data are presented as mean ± SD except for fear conditioning, where data are presented as mean ± SEM. *$p < 0.05$, **$p < 0.01$, ***$p < 0.001$, ns = not significant.

distance mice traveled (Figure 5E) was similar between vehicle-only Cps1 wild-type control (*Cps1*$^{flox/flox}$ [black circles]) and AAV8.CPS1-treated mice (blue triangles) (63.792 ± 8.895 m vs. 61.506 ± 6.454 m in male mice [p = 0.7767] and 68.626 ± 12.381 m vs. 63.135 ± 14.231 m in female mice [p = 0.3501]) over 30 min indicating similar locomotion activity. However, measurement of entry to the center during the first 5 min (Figure 5F), a sign of lower anxiety-like behavior, was reduced in AAV8.CPS1-treated mice (males, 25.625 ± 8.123 center entries vs. 8.067 ± 3.055 center entries, [p = 0.0025]; Cps1 wild-type control vs. AAV treated respectively; females, 24.875 ± 4.643 center entries vs. 12.875 ± 9.387 center entries, [p = 0.0035]; control vs. AAV treated respectively) who spend more time in the periphery than the center (Figure 5G) (purple = periphery; green = center), indicating an anxiety-like phenotype. In addition, the average speed of AAV8.CPS1-treated mice (blue triangles) was reduced compared to Cps1 wild-type controls (black circles) (Figure 5H) (males, 0.059 ± 0.009 m/s vs. 0.044 ± 0.002 m/s; control vs. AAV treated respectively; p = 0.0464; females, 0.061 ± 0.011 m/s vs. 0.037 ± 0.013 m/s, Cps1 wild-type control vs. AAV treated, respectively; p = 0.0001).

Viral characterization demonstrates a high percentage of partially full AAV capsids and fragmented genomes with 5′ truncations

Transmission electron microscopy (TEM) was performed with non-overlapping regions quantitated. On average, visualized fields demonstrated 73% full, 21% partial, and 7% empty capsids (Figure 6A). The size of the vector DNA packaged into AAV capsids was itself analyzed by alkaline gel electrophoresis of purified vector genomic DNA. Analysis of AAV8.Null (Figure 6B) demonstrated the two major vector sizes: dimer genomes at ∼3.6 kb (arrow) and monomer genomes at ∼1.8 kb (arrowhead). At 1.5 kb and smaller, fragmented genomes were visualized (at white asterisk [*]). By comparison, AAV8.CPS1 (Figure 6C) demonstrated a monomer genome sized at ∼5.2 kb (arrow) with a much more apparent and extensive size range of fragmented genomes (at black asterisk [*]).

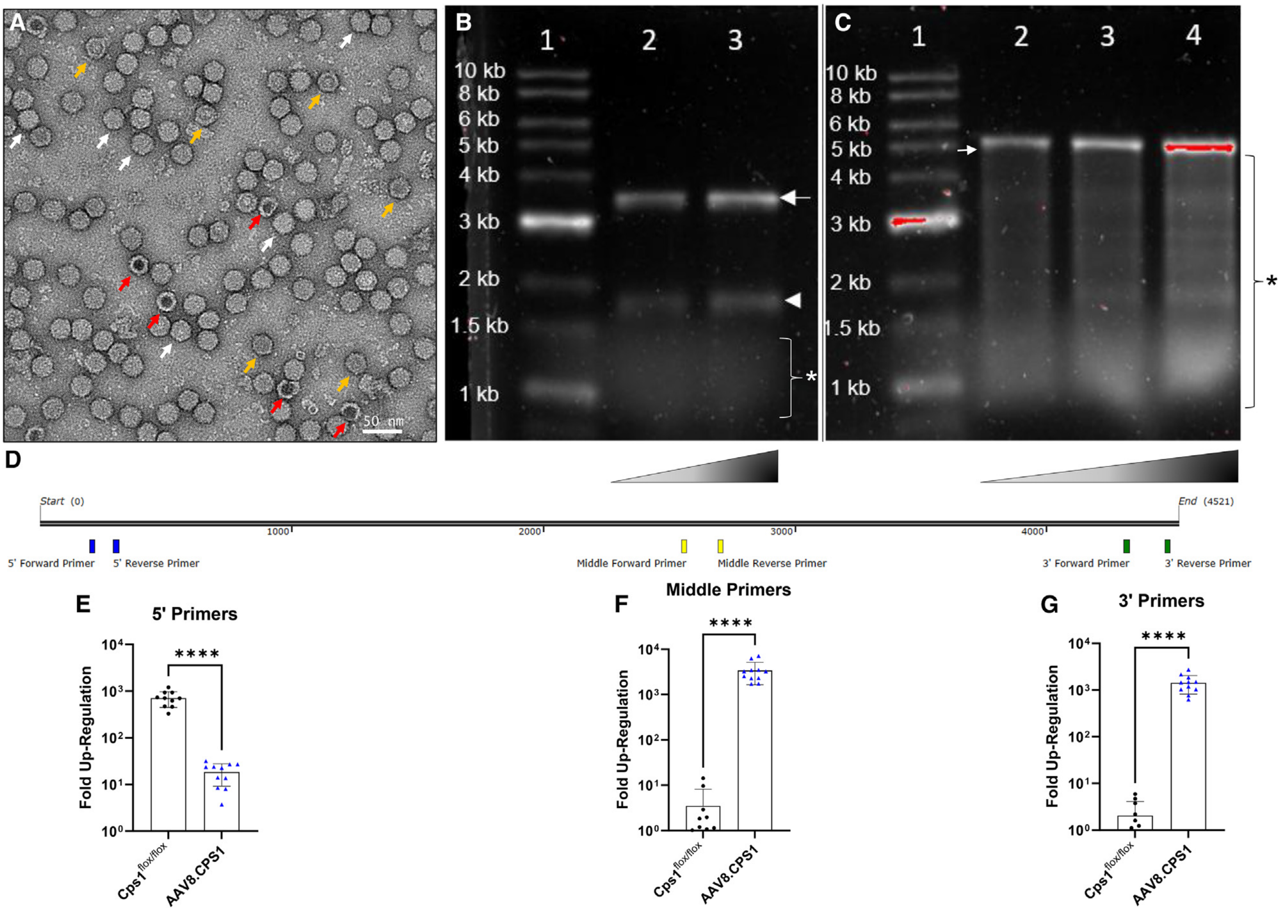

Figure 6. Oversized CPS1 AAV vector results in partial genome encapsidation and genome fragmentation

Final preparation of peak fractions of iodixanol ultracentrifugation purified AAV8 encapsidating the oversized *CPS1* AAV genome were stained in 2% uranyl acetate and imaged by TEM (A). The white arrowheads show examples of a full capsid as a genome containing AAV8.CPS1. The red arrowheads show empty AAV8 particles. The yellow arrowheads show AAV8 particles that are partially filled (scale bar, 50 nm). Genomes were extracted from a purified AAV8 vector preparation and the DNA was run on an alkaline agarose gel and detected by SYBR green staining. Size markers (1–10 kb) are shown on the left. Alkaline gel electrophoresis of AAV8.Null vector (B) demonstrates dimer genomes at ~3.6 kb (arrow) and monomer genomes at ~1.8 kb (arrowhead) (lane 1, 1 kb ladder; lane 2, 8.71 x 10^{10} genome copies; lane 3, 1.31 x 10^{11} genome copies), while AAV8.CPS1 (C) demonstrated a monomer genome sized at ~5.2 kb (arrowhead) with an extensive size range of fragmented genomes (at asterisk [*]) (lane 1, 1 kb ladder; lane 2, 4.36 x 10^{10} genome copies; lane 3, 8.71 x 10^{10} genome copies; and lane 4, 1.31 x 10^{11} genome copies.) The smearing is evidence of partial, fragmented genomes of various sizes. Cleaved heterogeneous genomes packaged into other capsids likely result in the large smear. Location of primer sets as related to CPS1 cDNA (D). Fold upregulation at 5′ end (E), middle of *CPS1* (F), and 3′ end (G). CPS1, carbamoyl phosphate synthetase 1. Data are presented as mean ± SD (*$p < 0.05$, **** $p < 0.0001$).

To in part determine where AAV genome truncation was primarily occurring, we designed three qPCR primer sets in the *CPS1* cDNA (Figure 6D) and performed quantitative real-time PCR (normalized to GAPDH expression) of reverse transcribed/cDNA synthesized from livers of mice (n = 10, Cps1 wild type; n = 11 AAV8.CPS1; n = 3, AAV8.Null) 9 months after AAV8.CPS1 vector administration. While primers based in the 5′ region of the *CPS1* cDNA demonstrated low levels of *CPS1* transgene expression (Figure 6E), primers in the middle (Figure 6F) and at the 3′ end (Figure 6G) displayed *CPS1* transgene expression up to 3 logs higher in the AAV8.CPS1-treated mice than in the Cps1 wild-type mice. These data demonstrate that AAV genome truncations primarily occurred on the 5′ genomic end.

To further characterize the packaged AAV8.CPS1 vector genome population, high-fidelity genome analysis on our research-grade iodixanol-purified AAV was performed. The basic schema for sequencing library preparation and EPI2ME wc-aav-qc analysis workflow is demonstrated in (Figure 7A). Viral DNA was extracted from AAV8.CPS1 lysates using Invitrogen PureLink Viral RNA/DNA Mini kit (Invitrogen, Carlsbad, CA; 12280050) then barcoded and sequenced using the Oxford Nanopore Technologies (ONT) Ligation Sequencing Kit (SQK-LSK114) on a single PromethION Flow Cell. The resulting full-length viral genome fastq file was used as the template. Additional input data consisted of the plasmid sequence of AAV8.CPS1, the host (HEK293 cell line) genome, the AAV8 packaging "rep-cap" plasmid sequence, and the AAV helper

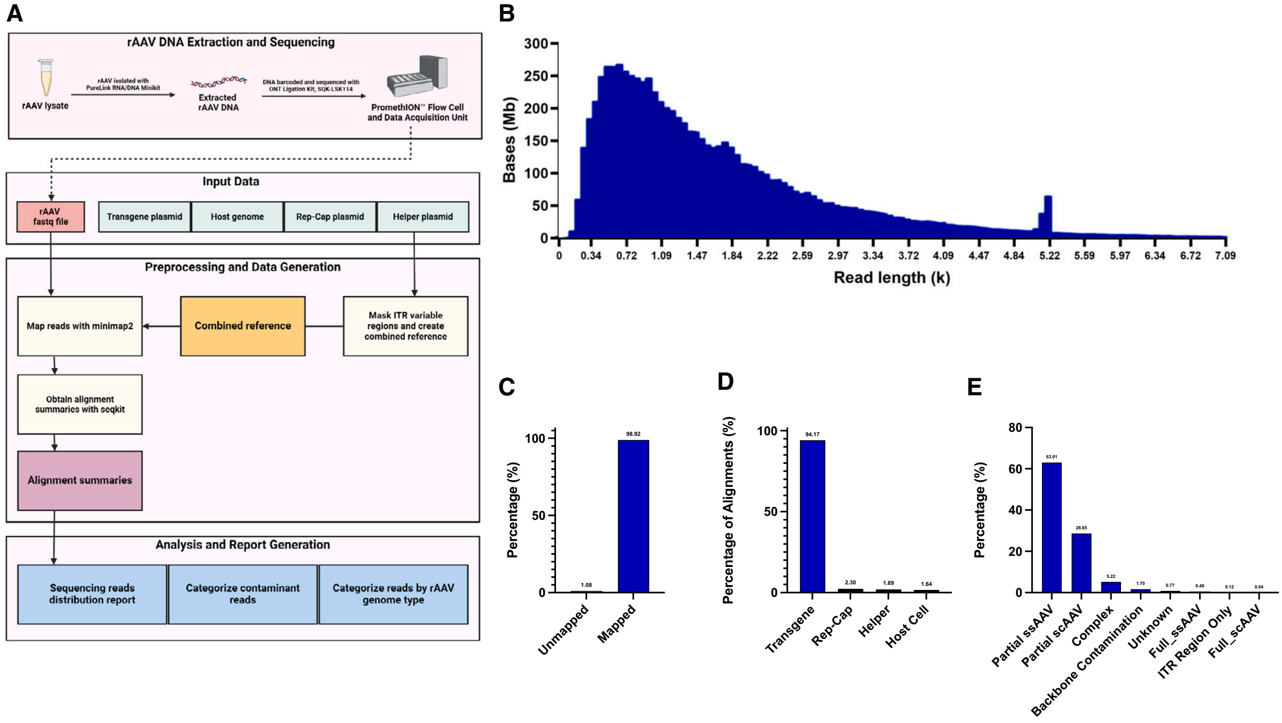

Figure 7. Oversized AAV8.CPS1 genome length fragmentation analysis

Basic schematic for sequencing library preparation, sequencing and EPI2ME, wc-aav-qc analysis workflow (A). AAV8.CPS1 was barcoded and sequenced using Oxford Nanopore Ligation Sequencing Kit, SQK-LSK114, on a single PromethION Flow Cell. Read distributions show a clear peak between 4.97 and 5.28 kb and a range of fragments indicative of truncated genomes and potential contamination (B). 98.92% of fragments aligned to the created reference sequence (combination of host, helper plasmid, packaging Rep-Cap plasmid, and transgene plasmid sequences) (C) with a vast majority of alignments (94.17%) mapping to the targeted transgene sequence (D). The percentages of the various AAV transgene genome types indicate the AAV8.CPS1 fragments identified in the sample (E). Data are presented as means. (A) Created with BioRender.com.

plasmid sequence, which was further processed and merged to create the custom combined reference. Reads from the sample viral fastq file were then mapped to this combined reference and alignment summaries were provided using the SeqKit fastq manipulation toolkit.[28] A sequencing reads distribution report was generated in addition to a description categorizing reads by contaminant and by recombinant AAV (rAAV) genome type.

Read distributions show a clear peak between 4.97 and 5.28 kb, as expected for the AAV8.CPS1 size, and a broad range of smaller genomic fragments indicative of truncated genomes (Figure 7B). 98.92% of encapsidated AAV fragments aligned to the previously described combined reference (Figure 7C) with the vast majority (94.17%) mapping to the targeted transgene plasmid (i.e., AAV8.CPS1) sequence (Figure 7D). Host cell genomic encapsidation was low (1.64%) as was rep-cap (2.30%) and helper (1.64%) genomic material packaging. The percentages of the various AAV transgene genome types (Figure 7E) indicated that AAV8.CPS1 fragmented genomes (i.e., partial single stranded AAV [ssAAV]) was the dominant genomic form (63.01%). Conversely, full-length single-stranded AAV ("full ssAAV") encompassing the complete genome cassette and ITRs was quite limited at 0.49%. There were almost no vector sequences detected >5.2 kb, suggesting that the upper packaging limit for AAV8 appears to be ~5.2 kb, similar to other AAV serotypes. Taken together with the RT-PCR data, the findings suggest that the CPS1 vectors produced tend to have intact 3′ ends but truncated 5′ ends and that these truncations occurred at many locations <5.2 kb from the 3′ end of the genomic cassette. Analysis of AAV8.Null is presented in the supplemental information.

DISCUSSION

CPS1 deficiency with neonatal onset typically presents with elevated plasma ammonia that may have already progressed to hyperammonemic encephalopathy. Neonates surviving this initial episode remain at risk of neurological deficits from cumulative injury due to repetitive hyperammonemic crises unless ammonia levels are well controlled, which is not an easy feat. Definitive therapy, to undergo a complete hepatectomy of an anatomically normal liver followed by a successful orthotopic liver transplant, typically requires time for the child to grow and obtain medical stability. Clinical management until that time may require long-term hospitalization. While carglumic acid, approved for treatment of N-acetylglutamate synthase deficiency, can be used to treat a subset of CPS1-deficient patients, this is not a broadly applicable therapeutic option as it depends on a *CPS1*

mutation with residual enzyme activity.[10] Thus, there is substantial unmet need for new treatments, including the development of an effective gene-based therapy for this disorder.

In this investigation, we have developed and tested an oversized serotype 8 AAV vector to express CPS1 using small liver-specific regulatory elements, codon-optimized *CPS1*, and a synthetic polyadenylation signal in a lethal conditional murine knockout model of the disorder. These studies demonstrate that, while female mice overall fare better than males (and we have detected this previously with a CPS1 dual-AAV-vector approach[19]), survival can be attained in AAV-treated mice. No nitrogen scavengers were required for survival, and mice were maintained with conventional (20% protein) mouse chow (i.e., not protein reduced) and carglumic acid without amino acid supplementation. Ureagenesis of ∼10% of normal was achieved with hepatic expression occurring primarily in perivenous hepatocytes; there was no evidence of elevated ALT or liver pathology in AAV8.CPS1-treated mice. Blood ammonia, while elevated, was relatively stable throughout the lifespan of surviving mice (euthanized at 9 months after study began, length of the study). Plasma glutamine, known to correlate with plasma ammonia in patients with CPS1 deficiency, was elevated but stable when compared to control mice; alanine and asparagine, which generally do not show good correlation with glutamine,[29] were also increased. While there was a reduction in glycine, taken together, these findings indicate some persistent dysfunction of the urea cycle. Nevertheless, the cycle is functional as demonstrated by the response to exogenous loading of ammonium: plasma levels increased as expected at 20 min and began to decline by the 60-min time point, indicating function of the enzyme and of the urea cycle itself (i.e., ureagenesis). Weights, measured daily as a dimension of health, were stable throughout and mice were not sarcopenic, unlike AAV8.Null-treated mice where weight loss was substantial. While some phenotypic studies did reveal a mild alteration in contextual learning and evidence consistent with at least mild anxiety, mice were active and appeared of normal behavior in conventional cages, and testing by a battery of measures was overwhelmingly no different than control mice with normal Cps1 activity. Thus, overall, biochemical stability was achieved and ureagenesis established with a metabolic response to ammonium challenging.

Exact values for the amount of CPS1 protein per milligram of rodent or human hepatocytes are not readily available. However, an estimate can be made from existing data. In the rat liver, there is 19–22.4 mg of matrix protein per gram of liver[30] and CPS1 constitutes 22% to 26% of total matrix protein[31]; this represents ∼4.2–5.8 μg of CPS1 protein per milligram of liver. Having achieved with the oversized AAV vector human CPS1 protein of ∼0.37 μg per milligram total liver protein, the expression level is ∼6.3%–8.8% of wild type.

The goal of the vector development for this large cDNA was to limit the size of the AAV genome so it could incorporate the obligatory *CPS1* therapeutic gene sequence and all necessary transcriptional elements. Multiple groups have reported a range of maximum genome packaging capacity of recombinant AAV vectors: 5.2,[32] 5.6,[33,34] and up to 6.0 kb,[35] larger than the size of wild type at 4.7 kb[20,32,36] and >105% of the normal virus genome, which has generally been considered the limit in packaging capacity.[37] Other studies, though, have suggested that, while the packaging limit is 5.2 kb, generally ∼5-kb fragmented genomes are encapsidated with attempts at oversized vectors resulting in a heterogeneous and broad size distribution[32] as truncated genome fragments (or fragment AAV [fAAV]). The data presented in this manuscript support this latter report with a maximum genome packaging capacity for AAV8 being ∼5.2 kb with a large percentage of heterogeneous fAAV. Expression by fragmented oversized AAV vector transduction may require homologous recombination of fAAV by a Rad51c-dependent repair pathway.[36]

As a preferred vector for hepatocyte transduction, AAV genome capacity is much smaller than other viral approaches like lentiviral vectors and has been identified as a major limitation for disorders associated with larger genes.[32] However, multiple groups have been able to show that it is possible to package more than 5 kb, such as for factor VIII with therapeutic activity[34,38–40] and in mutations of the dysferlin gene.[41] Using smaller highly active *cis*-regulatory elements and the essential components of a polyadenylation signal to restrict cassette size may allow delivery for a larger DNA cargo. Due to the size of the *CPS1* cDNA, though, the AAV genome capacity is markedly limited, making this a significant challenge. CPS1 is a complex enzyme of 160 kDa being composed of N-terminal and C-terminal moieties with multiple domains and multi-active centers.[4] The gene is large (110 kb), consisting of a 4,500-nucleotide coding sequence in 38 exons as a 1,500-amino acid protein before cleavage of a 38-amino acid signal peptide, and is dependent on NAG for allosteric activation[4,42,43]; many of its lysine residues are acetylated.[44] This complexity has no clear nucleotide sequence that can be excised and retain unaltered function (unlike factor VIII, CFTR, or dystrophin) to reduce cDNA size. With this large and unmodifiable (other than codon optimization) cDNA, and with the constraints of AAV packaging, we prioritized choosing the smallest highly active regulatory sequences possible to constrict genome size of the expression cassette for the ability to obtain hepatic CPS1 expression.

The TTR promoter (235 bp) mediates hepatocyte-specific transcription of transthyretin, a serum carrier protein of thyroxine and retinol.[45] In studies conducted by Monahan[46] evaluating and comparing small promoters for liver-directed expression *in vivo*, TTR provided the highest expression and thus was incorporated into our vector. We added a 5′ liver-specific transcriptional *cis*-regulatory module containing evolutionarily conserved clusters of transcription factor binding-site motifs (HSCRM8, 71 bp)[47] to further increase hepatic expression. Previous work by others has demonstrated that the absence of a 5′ intron leads to reduced gene expression.[46] Thus, we incorporated a 108-bp intron 5′ to the *CPS1* cDNA. This intron[48] is part of more than 90 different AAV, expression, and cloning vectors (NCBI BLAST accessed 5-28-2024 1,557 h). Finally, a nominal synthetic polyadenylation signal based on the rabbit β-globin gene, including the minimum sequence elements AATAAA, GT-rich, and T-rich regions to sufficiently constitute a

poly(A) site, was included.[49] Together, it was our goal to create a highly active minimally oversized AAV8 vector to produce CPS1 hepatic expression with survival, ammonia control, and normal brain activity and behavior.

The generation of this vector demonstrated challenges that have reduced its efficacy and utility in its present form. This AAV8, produced under the same methods for non-oversized research-grade AAV (e.g., AAV8.Null), had an unconcentrated digital droplet PCR (ddPCR)-determined titer of ~10% of non-oversized constructs (data not shown), consistent with the findings of others.[36,37,50] While the percentage of empty capsids was low as determined through capsid appearance by TEM, the purified virus suffered from a high percentage (nearly 25%) of partially filled capsids. However, this TEM-based determination greatly underestimated the true partial nature of the genomes. Analysis of the AAV genomes, first by alkaline gel electrophoresis, demonstrated evidence of genome fragmentation and, more broadly, with long-read nanopore sequencing, revealed that genome fragmentation was extensive (partial single stranded and partial self-complementary making up > 91% of vectors) with the percentage of intact full-length genomes being quite low (<1%). While ddPCR did demonstrate an acceptable titer of the purified vector, the primers were based within the approximately central region of the *CPS1* cDNA; thus, the effective titer of intact full-length genomes is substantially less than the titer determined by ddPCR due to vector fragmentation. Although reasonable titers of the vector were obtained, the ratio between fAAV and complete intact genomes is extraordinarily high, suggesting that full-length genome packaging is quite inefficient and, in fact, rare due to the nature of the oversized genome. Instead, fAAV is a very large percentage of both partial and full capsids as determined by barcoding and sequencing. Nonetheless, acute ammonia-mediated hepatic encephalopathy was prevented with this oversized *CPS1*-expressing vector. This in itself serves as evidence of the biochemical success that was achieved with this vector but limits its potential clinical utility in its present form.

Outside of the genome fragmentation and thus reduction in effective full-length genome titer, the effectiveness of the vector may be in part limited due to the site of AAV-based expression of CPS1 in the murine liver. As demonstrated by immunohistochemical staining, the vast majority and most dense CPS1 expression was in perivenous hepatocytes. While the native enzyme is expressed pan-hepatically in the liver, the urea cycle occurs primarily in periportal hepatocytes[51] where, in these studies, expression was markedly limited. This AAV8.CPS1 vector may have proved more efficacious in biochemical control with portal administration transducing primarily periportal hepatocytes[52] instead of systemic venous administration, an area for future investigation.

In conclusion, these studies demonstrate that an oversized AAV8.CPS1 vector, when administered intravenously, can establish at least partial ureagenesis, ammonia control leading to long-term survival without protein restriction, reduction of plasma ammonia in response to an exogenous ammonium challenge, and, most importantly, the prevention of acute hyperammonemic encephalopathy and death. With the limitations in its biochemical efficacy and evidence of genome fragmentation, these studies do serve as a proof of concept for an oversized AAV vector in addressing this neurologically devastating disorder. This vector as produced would not be applicable in its present form for clinical translation primarily due to the substantial genome fragmentation present. While the promoter and enhancer are likely safe and efficacious for clinical translation, the small percentage of intact full-length vector genomes is limiting. One could consider methods to improve isolation of intact full-length encapsidated AAV genomes. While full but incomplete encapsidated genomes and full complete AAV genomes may have slight differences in their surface charge properties, this may be challenging to exploit using anion-exchange chromatography, one method of AAV purification. In addition, the costs to produce enough high-quality full-length vector with the present yield may be prohibitive. While evidence of feasibility has been established, future directions will examine aspects of this vector to determine if additional genome size reduction (such as with the use of a recently described highly active 184-bp intron-less micro-promoter[53]) can be achieved to reduce genome size and thus fragmentation, and yield greater homogeneity of the final vector preparation without substantial loss of CPS1 activity, to achieve improved ureagenesis, ammonia control, and survival while continuing to prevent acute hyperammonemic encephalopathy. In addition, the use of a hypomorphic Cps1 murine model that our group is presently developing may allow for testing without the need for cre recombinase to produce the phenotype.

MATERIALS AND METHODS

Animal care and mouse procedures

Mice were housed in a vivarium at UCLA and sustained according to the National Institutes of Health guidelines with temperature and humidity control. Experimental procedures were approved by and conducted according to the guidelines of the UCLA Chancellor's Animal Care and Use Committee (ARC). Mice were maintained on a 12-h light-dark cycle and had *ad libitum* access to standard chow (20% crude protein) (PicoLab Irradiated Rodent Diet 20, 5053, Lab Diet, Richmond, IN, USA) and water. Daily weighing of mice was typically performed between 8 and 10 a.m. *Cps1*$^{flox/flox}$ mice (C57BL/6 background) were developed as previously described[18] and used for all studies. Genomic DNA was prepared from ear clips by standard methods, and genotyping was performed by PCR amplification as described.[18]

By random selection, mice were assigned to either control or experimental group. Male and female mice were represented throughout the study. For injections, 3×10^{11} gc/g of AAV8.CPS1 or AAV8.Null was diluted in sterile pharmaceutical-grade 0.9% saline (Medline, Northfield, IL, USA; 5T-NFSF-10011) and injected by intravenous approach (retro-orbital) under isoflurane anesthesia. AAV8.TBG.Cre recombinase (2×10^{11} gc/mouse, a dose previously demonstrated to result in Cps1 deficiency in this model[18]) (Addgene, Watertown, MA, USA) was administered simultaneously with the AAV8.CPS1 or AAV8.Null. All mice were weighed daily and closely

monitored for any signs of deteriorating health. Ill-appearing mice were euthanized at a humane endpoint before expiring. Mice received carglumic-acid-supplemented drinking water and were administered a dose of 1,000 mg/L NCA (Sigma-Aldrich, St. Louis, MO, USA; C4375-10G) with 5% (w/v) dextrose (Sigma-Aldrich, G8270); water was checked daily and replaced on an as-needed basis. Scheduled day 0, 10, 21, and thereafter monthly blood sampling was obtained from the retro-orbital plexus under isoflurane anesthesia with plasma frozen immediately and stored at −80°C until analysis. Samples of liver were either snap frozen at the time of euthanasia and stored at −80°C until analyzed or were prepared for immunohistochemistry as described below.

Molecular cloning

The CPS1 AAV vector was generated by standard cloning procedures utilizing restriction enzymes from New England Biolabs (Ipswich, MA, USA). Full-length codon-optimized human *CPS1* (hcoCPS1) was synthesized by Blue Heron Biotech (Bothell, WA, USA). The vector contained AAV2 ITRs and a minimal liver-specific promoter (TTR in pCCVC as a generous gift from Denise Sabatino, Children's Hospital of Philadelphia). The plasmid vector linearized and human codon-optimized *CPS1* was inserted by Gibson assembly (NEBuilder, #E2621). The liver-specific enhancer HSCRM8[47] (a generous gift from Beat Thony, Zurich Children's Hospital, Switzerland) was inserted by assembly into the linearized hcoCPS1-containing plasmid. All plasmids were sequence verified (Laragen, Culver City, CA, USA) prior to submission to the University of Pennsylvania Vector Core for viral amplification and purification of serotype 8 vector.

The following is the sequence of the 108-bp 5′ intron[48]: AGGT AAGTGCCGTGTGTGGTTCCCGCGGGCCTGGCCTCTTTACGG GTTATGGCCCTTGCGTGCCTTGAATTACTGACACTGACATC CACTTTTTCTTTTTCTCCACAG.

The following is the complete human codon-optimized *CPS1* sequence: ATGCCTCAGATCATAAAGATGACCCGGATTCTTAC CGCATTCAAGGTTGTAAGGACCCTTAAAACCGGCTTCGGCT TTACTAACGTGACCGCACACCAAAAGTGGAAGTTTAGCAG GCCCGGAATTCGCCTCCTTAGTGTGAAAGCCCAGACCGCT CATATAGTCCTTGAAGACGGCACAAAAATGAAAGGGTACT CATTCGGCCATCCATCATCTGTAGCCGGTGAGGTCGTGTTC AATACTGGATTGGGGGGTTATCCCGAGGCCATAACAGACC CAGCTTATAAGGGCCAGATCCTGACCATGGCCAACCCAAT CATCGGGAACGGAGGTGCGCCGGATACAACTGCGTTGGAT GAGCTGGGACTGTCCAAGTACTTGGAGAGCAATGGAATTA AAGTTTCTGGACTGCTGGTACTGGACTACTCAAAGGACTA CAATCATTGGCTGGCCACCAAAAGTCTGGGGCAATGGCTG CAGGAGGAGAAGGTGCCAGCTATATACGGAGTTGACACTA GAATGCTTACCAAAATTATAAGAGACAAAGGTACTATGCT GGGAAAAATTGAGTTTGAAGGACAGCCCGTGGATTTCGTA GACCCTAATAAGCAGAATCTTATCGCCGAGGTGAGCACAA AGGACGTTAAGGTCTACGGAAAAGGAAATCCAACTAAGGT GGTGGCTGTTGATTGTGGCATTAAGAACAACGTGATCAGA CTGCTGGTGAAACGCGGAGCTGAAGTCCATCTTGTCCCAT GGAATCATGATTTTACGAAAATGGAGTATGATGGAATTCTC ATCGCCGGCGGACCAGGGAACCCAGCCTTGGCTGAACCCC TTATCCAAAACGTTAGAAAAATACTCGAATCTGATAGGAA AGAGCCCCTTTTTGGTATATCCACCGGAAACTTGATTACAG GCCTTGCTGCAGGGGCCAAGACATATAAGATGAGCATGGC AAACCGCGGACAGAATCAGCCCGTACTGAACATTACTAAT AAGCAGGCTTTTATCACCGCACAGAATCACGGTTACGCTC TCGATAATACGCTCCCTGCCGGCTGGAAGCCGCTCTTCGT TAACGTAAATGATCAGACAAACGAGGGAATAATGCACGAA TCCAAACCCTTCTTCGCCGTCCAGTTCCACCCTGAAGTCAC TCCAGGCCCTATTGACACAGAATATCTCTTTGACTCCTTCT TTAGCCTGATAAAAAAGGGGAAGGCCACCACCATAACGTC CGTCCTGCCTAAGCCAGCTCTCGTGGCATCAAGAGTAGAG GTCTCCAAAGTGCTCATACTTGGTAGCGGGGGACTGTCAA TCGGCCAAGCAGGCGAGTTCGATTACTCCGGAAGCCAAGC AGTTAAGGCTATGAAAGAAGAGAACGTTAAAACTGTGCTG ATGAATCCAAATATAGCCTCCGTGCAGACCAATGAGGTGG GTCTCAAGCAAGCAGATACTGTTTACTTTCTTCCAATTACC CCCCAATTCGTAACCGAAGTCATTAAGGCCGAGCAGCCTG ATGGATTGATCCTGGGTATGGGCGGACAGACTGCACTGAA TTGCGGAGTGGAGTTGTTCAAAAGGGGTGTGTTGAAGGAA TATGGAGTTAAGGTACTCGGCACCTCCGTTGAGAGCATCA TGGCGACCGAGGATAGACAGTTGTTCTCTGATAAACTGAA CGAGATTAATGAGAAGATCGCCCCCTCATTCGCCGTGGAG TCTATCGAAGATGCACTGAAAGCCGCTGATACGATTGGCT ATCCTGTAATGATAAGAAGCGCCTACGCCCTGGGTGGCCT GGGGTCTGGCATCTGCCCTAACCGAGAGACGCTGATGGAC CTCTCCACAAAAGCCTTCGCCATGACTAACCAGATTCTGGT AGAAAAATCCGTCACCGGCTGGAAGGAAATTGAATACGAA GTAGTAAGAGACGCTGATGACAATTGCGTCACAGTCTGCA ACATGGAAAACGTCGATGCGATGGGCGTGCACACCGGAGA TTCCGTCGTTGTGGCGCCAGCACAAACACTCTCCAATGCTG AGTTCCAGATGCTCAGAAGAACAAGCATTAACGTTGTGCG ACATCTTGGGATAGTTGGCGAATGTAACATCCAATTTGCAC TGCACCCAACTAGCATGGAATACTGCATTATCGAAGTGAA TGCGCGGCTGAGCCGAAGCAGCGCTCTCGCCAGCAAAGCC ACAGGCTACCCACTTGCCTTCATTGCCGCAAAGATTGCACT GGGCATTCCACTGCCTGAGATTAAGAATGTCGTAAGCGGG AAGACAAGCGCCTGTTTTGAACCTTCCCTGGACTATATGGT GACTAAGATTCCTCGGTGGGACCTTGATAGGTTCCATGGG ACCTCATCTaGAATAGGATCATCAATGAAGTCTGTGGGTGA AGTGATGGCTATCGGGCGGACCTTcGAaGAGAGTTTTCAGA AAGCACTTCGGATGTGTCACCCCTCAATTGAGGGCTTCACC CCCCGGTTGCCAATGAACAAGGAGTGGCCATCAAACCTGG ACCTGAGAAAAGAGCTCAGCGAGCCTAGCTCAACTAGAAT CTACGCAATCGCCAAGGCAATCGACGATAACATGTCATTG GATGAGATAGAGAAGTTGACATACATAGACAAATGGTTCC TCTACAAAATGCGAGACATTCTGAATATGGAGAAAACACT GAAGGGACTGAATTCTGAGAGCATGACGGAGGAGACACTT AAGAGAGCAAAAGAGATTGGGTTCAGCGATAAGCAAATTT CAAAGTGCCTTGGACTGACCGAAGCCCAGACACGGGAGCT GAGACTGAAGAAAAATATACACCCATGGGTGAAGCAGATC GACACCCTGGCGGCCGAATATCCCAGCGTTACTAATTACCT GTATGTTACATATAACGGCCAAGAGCATGACGTAAATTTTG

ACGATCATGGAATGATGGTTTTGGGATGCGGTCCCTACCACATTGGCTCTTCAGTGGAGTTTGATTGGTGCGCAGTGAGCTCCATTCGGACCCTCAGACAGCTTGGAAAAAAAACAGTGGTGGTAAATTGTAACCCGGAGACTGTGTCAACCGACTTCGACGAATGCGACAAGTTGTATTTTGAGGAATTGAGTCTTGAAAGGATTCTTGATATCTACCATCAGGAAGCATGCGGAGGCTGTATTATCTCAGTGGGCGGGCAGATACCCAACAACCTTGCTGTACCTCTCTATAAAAACGGTGTAAAGATCATGGGCACCTCTCCCCTCCAGATTGACAGGGCCGAGGACCGCTCAATTTTCAGTGCTGTGCTGGACGAACTCAAAGTCGCTCAAGCTCCTTGGAAAGCTGTTAATACTCTTAACGAGGCCCTCGAGTTCGCCAAGTCTGTGGATTACCCATGTCTTCTTCGGCCCTCCTACGTGCTGTCAGGATCCGCAATGAACGTCGTGTTCAGCGAGGATGAAATGAAGAAATTTCTGGAGGAGGCTACACGGGTGAGTCAAGAGCATCCTGTGGTTTTGACTAAGTTCGTTGAGGGCGCCCGGGAAGTCGAGATGGATGCAGTCGGTAAAGATGGACGGGTAATTAGCCACGCAATTAGTGAACACGTGGAAGATGCCGGGGTCCATTCTGGCGACGCCACTCTCATGCTGCCAACACAGACAATTAGTCAGGGTGCTATAGAGAAAGTGAAAGATGCGACTAGGAAGATCGCAAAAGCCTTCGCAATATCTGGCCCATTTAACGTGCAGTTTCTCGTGAAAGGTAACGACGTCCTGGTGATCGAGTGTAATCTCCGAGCGTCACGATCCTTCCCTTTCGTAAGCAAGACCCTCGGCGTAGACTTTATTGACGTGGCCACGAAAGTTATGATTGGAGAGAATGTAGACGAGAAACACCTCCCCACTCTTGACCATCCGATCATCCCCGCGGATTATGTTGCCATCAAGGCCCCAATGTTCTCTTGGCCGCGCCTGCGAGACGCTGATCCCATCTTGCGCTGTGAAATGGCAAGCACAGGCGAAGTAGCATGCTTCGGCGAAGGTATTCATACCGCATTTCTGAAGGCCATGCTGAGCACCGGCTTCAAGATCCCCCAGAAGGGTATCCTCATCGGCATCCAGCAGTCTTTCCGCCCAAGATTCCTGGGGGTAGCAGAACAACTTCATAACGAAGGCTTCAAGCTGTTTGCAACAGAAGCAACCTCTGATTGGCTGAACGCTAATAATGTTCCTGCGACTCCAGTCGCCTGGCCCAGCCAGGAAGGACAAAATCCCAGCCTGTCTAGCATCAGAAAACTCATACGAGATGGCTCTATCGACCTTGTTATCAACCTGCCTAATAACAACACCAAATTTGTCCACGACAACTACGTCATCAGAAGAACTGCCGTGGATAGCGGTATCCCCCTGCTGACCAATTTCCAGGTTACCAAGCTCTTTGCAGAAGCTGTTCAGAAATCTCGCAAGGTGGATAGCAAGTCACTGTTTCACTATCGACAATATTCAGCGGGGAAGGCTGCATAG.

The sequence of the synthetic polyadenylation signal was based on the essential components as described by Proudfoot[49]: AATAAAAGATCAGAGCTCTAGAGATCTGTGTGTTGGTTTTTTGTGT.

The plasmid pAAV.TBG.PI.Null.bGH was a gift from James M. Wilson (Addgene plasmid #105536; http://n2tnet/addgene:105536, RRID:Addgene_10536) and was used to produce AAV8.TBG.Null vector by similar methods.

AAV vector development

Recombinant AAV8 was produced at the University of Pennsylvania Vector Core (Philadelphia, PA) as previously described.[54] In brief, polyethylenimine as a transfection agent was used to transfect plasmids AAV *cis*, AAV *trans*, and adenovirus helper into HEK293 cells. Three days after transfection, culture supernatants were collected. AAV particles were subsequently purified by an ultracentrifugation iodixanol step gradient. Viral titering by genome copy number was performed by ddPCR. The primer/probe were designed to be in the middle of the hcoCPS1 gene, F-TCATTGCCGCAAAGATTGC; R-CCGCTTACGACATTCTTAATCTCA, FAM-CTGGGCATTCCACTGC-MGB.

TEM and full/partial/empty capsid ration determination

Ultracentrifugation iodixanol step-gradient purified final research-grade AAV preparation was used to determine full/partial/empty capsid ratio. AAV (2.5 μL) was placed on a continuous copper grid (Ted Pella, Redding, CA, USA; 01803-F) then stained with 2% uranyl acetate for contrast. TEM images were then acquired with 1-s exposure on an FEI Tecnai G2 transmission electron microscope (Hillsboro, CA, USA) to generate digital micrographs. Five images from separate, non-overlapping regions were examined and full/partial/empty capsid at 50 nm were determined by a single observer.

Alkaline gel electrophoresis

A 1% agarose alkaline gel was prepared by combining 0.5 g of agarose, 49 mL of 1× Tris-acetate-EDTA (TAE) buffer, and 1 mL of 50× alkaline gel buffer (2.5 M NaOH, 50 mM EDTA, pH 13–14). Once solidified, the gel was placed in a gel electrophoresis chamber filled with 1× alkaline running buffer (0.5 M NaOH, 50 mM EDTA).

500 μg of a 1-kb DNA ladder (Lamda Bio, Ballwin, MO, USA; M108-5) was diluted to 25 μL with sterile saline 0.9% (KD Medical, Columbia, MD, USA; RGC-3290). Purified AAV8 (TBG.Null and CPS1) corresponding to concentrations of 4.36×10^{10}, 8.71×10^{10}, and 1.31×10^{11} genome copies, respectively, were standardized to 16.5 μL with sterile saline. 8.5 μL of alkaline loading dye (80% 4× alkaline gel buffer, 20% glycerol, 0.12% SDS, 0.0001% xylene cyanol) was added to each ladder and virus aliquot and were heated at 95°C for 3 min. Samples were cooled on ice and loaded into the gel. The gel was run at 80 V and 400 mA for 3 h.

Once the run was complete, the gel was placed in a light-proof container with 100 mL of Tris-HCl (0.1 M, pH 8.5) and rocked on a shaker for 1 h at room temperature. The gel was then stained with 4× SYBR Safe DNA Gel Stain (Invitrogen, S33102) in 0.1 M NaCl and rocked on a room-temperature shaker for 2 h. The gel was rinsed with ddH_2O and shaken in ddH_2O for 5 min. The gel was visualized and imaged with a ChemiDOC XRS+ Molecular Imager (Bio-Rad, 170–8265).

AAV genome length determination

AAV8.CPS1 viral DNA was isolated for an iodixanol ultracentrifugation-purified preparation using PureLink RNA/DNA Minikit (Invitrogen, 12280050) following the manufacturer's instructions. The starting material for genome isolation was $>2.6 \times 10^{10}$ genome copies of AAV8.CPS1. Following ONT (Oxford, UK) instructions, the standard ligation sequencing protocol was followed; ONT recommended

a minimum of 1 μg of starting material for ligation sequencing when expecting an N50 of 10 kb. The ONT PromethION device was used to produce long nanopore sequencing reads for downstream determination of full-length, native rAAV genomes for both single-stranded and self-complementary viral vectors. Minimum read lengths were reduced to 20 bp and a high-accuracy (HAC) basecalling model was used. The final sequence run summary report includes total number of bases (Mb) compared to read length in thousand base pairs (k). The EPI2ME solution workflow (ONT, Oxford, England), wf-aav-qc, was then used to analyze and identify genome fragments.[55] Input data consisted of the sequenced AAV8.CPS1 fastq file and a custom merged reference sequence, consisting of the plasmid sequence of AAV8.CPS1, the HEK293 cell genome (GRCh38, https://www.ncbi.nlm.nih.gov/geo/query/acc.cgi GEO: GSM5266682), the AAV8 packaging rep-cap plasmid sequence (Addgene plasmid 112864), and the AAV helper plasmid sequence (Addgene plasmid 112867). The sample genome was then aligned to the described combined reference sequence in order to determine amount of reads mapping to specific genomes. Downstream analyses included frequency of start and end positions of alignments within the transgene plasmid ITR-ITR region in order to identify potential truncation hotspots and description of AAV structures. The wf-aav-qc pipeline was applied with an increase of the default "itr_fl_threshold" (ITR full-length threshold) parameter by 10-fold in order to compensate for mutations found within the ITR sequences.

Routine, special staining, and immunohistochemistry

Whole livers were removed from euthanized mice after transcardiac perfusion with cold PBS. Livers and brains underwent fixation in 10% neutral buffered formalin and were stored in 70% ethanol until embedding in paraffin. Blocks were sectioned at 4-μm thickness onto glass slides. H&E staining and trichome were performed by standard techniques.

Immunostaining was performed on a Leica Bond RX auto-stainer using Bond refine kit (Leica Biosystems, Nussloch, Germany, DS9800). Briefly, Leica Bond RX routine factory based "Bake and Dewax" protocol was followed. Heat-induced antigen retrieval using ER1 (BOND Epitope Retrieval Solution 1, Leica Biosystems, AR9961) buffer was performed for 20 min followed by a peroxide block for 5 min. This was followed by Bond Wash buffer three times for 0 min each. Primary antibody CPS1 (Abcam, Milpitas, CA, USA; AB45969 1-1000) was applied for incubating for 1 h, then Bond Wash buffer (Leica Biosystems) three times for 0 min each. Incubation with Dakocytomation Envision System labeled polymer horseradish peroxidase (HRP) anti-rabbit (Agilent, Santa Clara, CA; K4003, ready to use) for 10 min followed. Bond Wash buffer was performed five times 0 min each followed by deionized water wash for 0 s. This was followed by incubating with Mixed DAB Refine (Leica Biosystems) for 10 min. Next, slides were washed with a deionized water wash three times for 0:00 s each. Slides were counterstained by incubating with hematoxylin for 10 min and followed by Bond Wash buffer three times for 0 s each. Slides then were rinsed with deionized water dehydrated in series of alcohols, cleared with Histo-clear (VWR, Radnor, PA, USA; 64110) and mounted with Permount (Fisher Scientific, Waltham, MA; SP15-100).

Plasma ALT determination

ALT was determined on plasma from venous blood collected by capillary tubes with sodium heparin (Globe Scientific, Mahwah, NJ, USA; 56186). Plasma was collected and stored at −80°C until ready for processing, which was performed by IDEXX Veterinary Services reference laboratory (Westbrook, ME, USA).

qPCR

Tissue samples were collected from mice, and total RNA was extracted from livers using the RNeasy Fibrous Tissue Mini Kit (Qiagen, Hilden, Germany; 74704) according to the manufacturer's instructions. 1 μg of RNA was reverse transcribed using the High Capacity cDNA Reverse Transcription Kit (Applied Biosystems, Waltham, MA, USA; 4368814). Following cDNA generation, real-time PCR (qPCR) was performed using SsoAdvanced Universal SYBR Green Supermix (Bio-Rad, Hercules, CA, USA; 1725274) in order to quantify the expression of the *CPS1* and *GAPDH* genes using three sets of primer pairs toward the 3′, middle, and 5′ sections. Melting temperatures for the primer sets were 54°C, 56°C, and 56°C, respectively. Quantification was determined via threshold cycle (C_T) values using the MyiQ2 Two Color Real-Time PCR Detection System (Bio-Rad, 170-9790). The qPCR protocol was done as follows: DNA denaturation was done for 3 min at 95°C, primer annealing was done for 10 s at 95°C, then primer extension was done for 25 s at 56°C. The primer annealing and primer extension steps were repeated for up to 40 cycles. *CPS1* expression was normalized to endogenous *GAPDH*, and fold enrichment of the AAV8.Null and AAV8.CPS1 groups was calculated using the $2^{-\Delta\Delta Ct}$ method in comparison to the Cre-only control group. Primer sequences were as follows: 5′ *CPS1* primers (IDT) amplify a 114-bp region: *CPS1* F (binds bases 198–217): CTTTGGCCATCCCTCCT CTG, *CPS1* R (binds bases 292–311): TTGGCCATGGTGAGGAT CTG. Middle *CPS1* primers (IDT) amplify a 165-bp region: *CPS1* F (binds base pairs 2548–2567): AGCGAGCCTAGCTCAACTAG, *CPS1* R (binds base pairs 2693–2712): GCTCTCAGAATTCAGT CCCT. 3′ primer pair (IDT) amplifies a 183-bp region: *CPS1* F (binds base pairs 4307–4327): GCTCTATCGACCTTGTTATCA, *CPS1* R (binds base pairs 4470–4489): AGTGAAACAGTGACTTGCTA. *GAPDH* primers (Invitrogen, 10336022): *GAPDH* F: AGCCACA TCGCTCAGACAC, *GAPDH* R: CGCCCAATACGACCAAATC.

In situ hybridization (RNAscope)

RNAscope *in situ* hybridization was performed using Bond RX platform (Leica Biosystems) and RNAscope 2.5L reagent kit (Advanced Cell Diagnostics [ACD], Newark, CA, USA; 322750) according to the manufacturer's protocol (document number: 322750-USM). Briefly, freshly cut 4-μm-thick paraffin sections were treated with heat-induced epitope retrieval (HIER) (ACD HIER 15 min with ER2 at 95°C) and proteinase digestion (ACD 15 min protease). The slides were then incubated for 2 h at 40°C with hCPS1-codon-No-XMm (ACD-ref. 1322848-C1) targeting 2–1,687 of hcoCPS1 sequence. Amplification steps were performed according to the

ACD protocol. The chromogen was detected with the ACD RNAscope 2.5LSx Reagent Kit-RED (ACD, 322750). Slides were scanned at high magnification (×400) using a whole-slide scanning microscope (Aperio, Leica Biosystems).

Western blot

Unfixed frozen liver (stored at −80°C until use) was homogenized with soluble protein isolated in radioimmunoprecipitation assay (RIPA) buffer (Thermo Fisher Scientific, 89900) and HALT protease inhibitor (Thermo Fisher Scientific, 78430). Quantification of protein was performed using Bio-Rad protein assay dye (Bio-Rad, 5000006). Protein was loaded into a 12% gel (Bio-Rad, 4561045) followed by transfer to polyvinylidene fluoride (PVDF) membranes using the TransBlot Turbo system according to the manufacturer's instructions (Bio-Rad, 1704156). Membranes were blocked in 5% milk before incubation with primary antibodies overnight at 4°C with gentle agitation. Membranes were incubated with secondary antibodies for 1 h at room temperature with gentle agitation. Due to the marked differences in abundance of carbamoyl phosphate synthetase 1 protein as Cps1 in wild-type mice and vector-derived CPS1 in AAV8.CPS1-treated mice, antibody concentrations were adjusted so that bands could be visualized on the same gel. Primary antibodies for $Cps1^{flox/flox}$ (i.e., wild-type mice): anti-CPS1 (Invitrogen, PA576339, used at 1:5,000); anti-β-actin (Santa Cruz Biotechnology, Santa Cruz, CA; catalog no. SC47778, used at 1:5,000). Primary antibodies for AAV8.CPS1: anti-CPS1 (Invitrogen, PA576339, used at 1:500); anti-β-actin (Santa Cruz Biotechnology, Santa Cruz, CA; catalog no. SC47778, used at 1:5,000). Secondary antibodies: goat anti-rabbit IgG H&L (HRP) (against CPS1 primary; Abcam, ab205718, used at 1:5,000) goat anti-mouse IgG H&L (HRP) (against β-actin primary; Bio Rad, 1706516, used at 1:5,000). Detection was performed using SuperSignal West Pico PLUS Chemiluminescent Substrate (Thermo Fisher Scientific, 34577). Blots were imaged using the iBright FL1500 imaging system (Invitrogen, A44241).

CPS1-specific quantitative ELISA

AAV vector-derived human CPS1 protein was quantified using a custom-prepared ELISA from LS Bio (LS-F66674, Newark, CA, USA). Unfixed frozen liver (stored at −80°C until use) was homogenized with soluble protein isolated from terminal time-point mice ($N = 10$, both sexes represented). Samples were quantitated in duplicate following the manufacturer's instruction.

Ammonia challenge

Mice underwent ammonia challenging at the same time of the day. To simulate a nitrogen challenge, mice were injected by intraperitoneal method with a 4-mmol/kg solution of $^{15}NH_4Cl$ (Cambridge Isotope Laboratories, Andover, MA, USA; NLM-467-1). Fifteen minutes after injection, the mice were evaluated behaviorally by scoring from one observer using the scale outlined in Ye et al.[56] Blood was collected at the indicated time points after injection. Whole-blood ammonia was determined (with dilutions as needed) using the Arkray PocketChem BA meter (Arkray, Kyoto, Japan).

Ureagenesis

1-^{13}C-sodium acetate (Cambridge Isotope Laboratories, Andover, MA, USA; CLM-156-1) as a 1% solution prepared in filter-sterilized PBS was administered by intraperitoneal method. Thirty minutes after tracer was injected, blood was collected with lithium heparin capillary tubes (Globe Scientific) and plasma isolated for ureagenesis rate assessment. Samples were shipped to Metabolic Solutions (Nashua, NH, USA) for rate analysis by isotopic ratio mass spectrometry.

The ^{13}C urea enrichment was determined by converting urea to a trimethylsilyl (TMS) derivative. Briefly, 25μL of plasma was deproteinized with 250 μL of acetonitrile and centrifuged at ambient temperature for 10 min. The supernatant was then poured into a correspondingly labeled screw-cap culture tube and evaporated to dryness at 60°C using nitrogen gas. Pyridine (50 μL) and N-methyl-N-(t-butyldimethylsilyl) trifluoroacetamide (MTBSTFA) was added to the dried sample. Reagent and sample were heated for 30 min at 60°C ± 10°C. After the reaction was complete, the sample was transferred to an autosampler vial for analysis. Controls and samples were analyzed using a Thermo Fisher Delta V gas chromatograph combustion isotope ratio mass spectrometer. A Phenenomenex (Torrance, CA, USA) ZB-5 plus capillary column 30 m × 0.25 ID (mm) × 0.50 film thickness (μm) was used for chromatography of urea-TMS derivatives. Gas chromatography conditions were at an initial temperature of 120°C, then ramped at 15 °C/min/min to 240°C. The ^{13}C urea enrichment was converted to atom % ^{13}C by common conventions. The atom % ^{13}C excess (APE) was adjusted for the natural background level of ^{13}C in urea prior to isotope administration plus adjusted by the factor 13 due to the number of added carbon atoms in the TMS derivative.

The urea concentration was similarly determined by converting urea to a TMS derivative in a separate analysis but with the addition of 25 μL of internal standard, $^{15}N_2$,^{13}C-urea (Cambridge Isotope Labs) to 25 μL of plasma. The urea concentration was determined by linear regression of the measured area ratios of urea versus internal standard against urea standards in water of known concentration. Standards, controls, and samples were analyzed for urea and $^{15}N_2$,^{13}C-urea by gas chromatography-mass spectrometry (GC-MS) using electron ionization (Agilent 5977B GC/MS) and selected ion monitoring for m/z 231 and m/z 234, respectively. Similar chromatography conditions as described above were used for the urea concentration measurement.

Ureagenesis was calculated as the multiplication of APE urea values by urea concentration in plasma yielded the absolute concentration (μM) of newly formed ^{13}C-labeled urea.

Amino acid and ammonia analysis

The concentration of amino acids in plasma was determined by cation exchange chromatography.

(1) Plasma sample preparation

Plasma samples were vortexed with 5-sulfosalicylic acid (2% w/v) as deproteinization agent, lithium citrate loading buffer pH 2.20 (Biochrom US, Harvard Bioscience, Holliston, MA, USA) (62.2% v/v) as a diluent and pH regulator, glucosaminic acid, as an internal standard, and high-performance liquid chromatography (HPLC)-grade water. Samples were spun for 5 min at 900 × *g* with a mini-centrifuge (SARSTEDT AG, Newton, NC, USA). Supernatant was filtered with 0.2-μm filter (Celltreat, Pepperell, MA, USA).

(2) Plasma amino acid analysis

Forty-two amino acids and ammonia were analyzed per sample, using the Biochrom 30+ amino acid analyzer and Midas autosampler (Biochrom US, Harvard, Holliston, MA). Injected sample entered a polyether ether ketone (PEEK) lithium ion-exchange column using lithium citrate buffers at varying pH (2.80–3.55) and increasing molarity at defined temperature gradient and flow rates, according to the manufacturer's standard protocol. Ninhydrin (Fujifilm Wako Chemicals, Richmond, VA) then reacted with column eluent, and absorbances were continually registered at 440 and 570 nm. Each amino acid was determined by retention time. Concentrations were calculated (OpenLAB CDS EZChrom software) through integration of peaks in relation to authentic standards (MilliporeSigma, Burlington, MA), plus asparagine, glutamine, homocysteine, tryptophan, alloisoleucine, and homocitrulline) (individually, from Millipore Sigma), using calibration curves with $r^2 \geq 0.99$.

Behavioral testing

Behavioral tests were performed during the light cycle (06:00–18:00) between the hours of 12:00 and 17:00. Mice were handled by the experimenters for 5 days prior to any behavioral testing to reduce handling stress during testing. All mouse home cages were placed in the behavior room at least 1 h prior to testing to acclimate them to the environment. Experimenters were blinded to the treatment conditions.

(1) SHIRPA primary screen

SHIRPA was developed as a standardized set of experimental procedures to characterize the phenotype of genetically modified mice.[57] The protocols were designed to test muscle function, cerebellar function, sensory function, and neuropsychiatric function. The SHIRPA Primary screen was performed as previously described.[58] Mice were observed for behaviors and responses to a series of manipulations. Measures of activity levels, autonomic responses and reflexes, gait and locomotion, balance, coordination, muscle tone, strength, fear, and aggression were all assessed quantitatively. Assessments were done in a viewing cylinder, open arena, on a grid, and while held by the tail or restrained in a supine position.

(2) Open field and NOR

The open-field and NOR tests were performed as previously described.[59–61] The apparatus consisted of 40 × 40-cm gray acrylic plexiglass with 40-cm-high walls (made in house). All testing was performed under low lighting (<20 lux). On day 1, mice were habituated to the empty open field and allowed to explore for 30 min. This habituation session was scored for overall locomotor activity, as well as anxiety-like behavior in the first 5 min (i.e., time and number of entries into the center of the open field). Twenty-four hours later, NOR training was performed with two identical objects placed in opposite corners of the field and mice were allowed to explore for 10 min. After a 30-min delay, one of the objects was replaced with a novel object and mice were again allowed to explore for 10 min for the testing phase. The discrimination index was calculated for the testing phase ([novel object exploration time − familiar object exploration time]/total exploration time). Any mice that did not reach the minimum exploration time of 20 s for both objects during either the training or testing phase were excluded from analysis. The objects, combinations of objects, and locations were used in a balanced manner to reduce potential bias. All sessions were recorded by overhead video and analyzed using AnyMaze software (Stoelting, version 7.3, Wood Dale, IL, USA).

(3) Contextual fear conditioning

Contextual fear conditioning is a test of associative learning primarily focused on hippocampus functioning with the involvement of the basolateral amygdala.[62–64] Mice were placed into standard conditioning chambers with metal-grid floor that reside within sound attenuating chambers (MedAssociates, Fairfax, VT). Mice underwent pre-exposure to the context for 10 min to reduce baseline freezing. Twenty-four hours after the pre-exposure, subjects were placed back in the conditioning chamber for 3 min before the onset of the first foot-shock unconditional stimulus (US; 0.75 mA, 2 s). Animals were exposed to three USs in total, with an inter-trial interval of 1 min. After the third US, the mice were left in the conditioning chamber for another 60 s, at which point they were retrieved from the conditioning chambers and then placed back in their home cages. Twenty-four hours later, the mice were returned to the same context as they were trained in and recorded for 8 min. No shocks were given during the test session. The Video Freeze software (MedAssociates) was used to analyze the amount of movement to assess levels of freezing as a measure of fear of the context. A freezing threshold of 19 was set as the threshold to detect freezing based on prior hand-scoring data obtained by the lab.

Statistical analysis

Collected data were analyzed with a statistical software package (GraphPad Prism 10 Software, San Diego, CA). Results were expressed as mean ± standard deviation (SD) except in fear conditioning, where it was expressed as standard error of the mean (SEM). p values were determined using one-way ANOVA with Tukey's multiple comparison's test, or unpaired t test with Welch's correction when applicable. Error bars represent SD or SEM as indicated. $p < 0.05$ was considered significant. For behavioral testing, NOR, and open-field test, two-way ANOVA; for fear conditioning, three-way ANOVA; and for SHIRPA, one-way ANOVA.

DATA AVAILABILITY

Plasmids for vectors will be made available to those who have a legitimate request unless they originate from another investigator or commercial entity whereby those individuals/companies can be contacted directly. The data that support the findings of this study are available from the corresponding author, G.S.L., upon reasonable request.

ACKNOWLEDGMENTS

These studies were funded by an R61 grant (R61NS121348) (G.S.L.) from the United States National Institutes of Health/National Institute of Neurological Disorders and Stroke (NINDS) and the Swiss National Science Foundation grant 320030_207965 (J.H.). Research reported in this manuscript was also supported by NICHD of the National Institutes of Health under award number 5P50HD103557.

Drawings (mouse in Figure 1) were developed by Sarah Pyle at Sarah Pyle Design (San Francisco, CA) and BioRender (Toronto, Ontario, Canada) (graphical abstract and Figure 7 (Diep, T.; 2025) (https://BioRender.com/q14s417]). The authors thank David Strugatsky PhD from UCLA for assistance with TEM and Kenton Woodard PhD from the University of Pennsylvania Vector Core for helpful discussion regarding the vector genome analysis. For TEM, data were acquired at the Electron Imaging Center for Nanosystems (EICN) at the University of California, Los Angeles's California for NanoSystems Institute (CNSI). Appreciation to Casey Hanson PhD, computational lead, and Benjamin Bastin for the sample processing and sequencing data acquisition of AAV8.CPS1, both of the Applied Genomics, Computation, & Translational Core at Cedars-Sinai Medical Center.

AUTHOR CONTRIBUTIONS

Experimental design, data acquisition, interpretation, analysis, manuscript preparation, G.S.L. Data acquisition, analysis, critical review of the manuscript, T.D., W.Z., L.L., and R.E.R. Data acquisition and critical review of manuscript, M.N. and J.H. Data acquisition, I.L.D., G.M., I.Z., J.G., H.P., B.A.B., Y.L., and S.B.

DECLARATION OF INTERESTS

G.S.L. serves as a consultant to Astellas Gene Therapies and has received grant support from the Association of Creatine Deficiencies in an area unrelated to the work described in this manuscript. The Regents of the University of California have submitted a patent application for this work.

SUPPLEMENTAL INFORMATION

Supplemental information can be found online at https://doi.org/10.1016/j.omtn.2025.102470.

REFERENCES

1. Nitzahn, M., and Lipshutz, G.S. (2020). CPS1: Looking at an ancient enzyme in a modern light. Mol. Genet. Metab. *131*, 289–298. https://doi.org/10.1016/j.ymgme.2020.10.003.
2. Nyunoya, H., Broglie, K.E., Widgren, E.E., and Lusty, C.J. (1985). Characterization and derivation of the gene coding for mitochondrial carbamyl phosphate synthetase I of rat. J. Biol. Chem. *260*, 9346–9356.
3. Summar, M.L. (1998). Molecular genetic research into carbamoyl-phosphate synthase I: molecular defects and linkage markers. J. Inherit. Metab. Dis. *21*, 30–39. https://doi.org/10.1023/a:1005349306311.
4. Haberle, J., Shchelochkov, O.A., Wang, J., Katsonis, P., Hall, L., Reiss, S., Eeds, A., Willis, A., Yadav, M., Summar, S., et al. (2011). Molecular defects in human carbamoy phosphate synthetase I: mutational spectrum, diagnostic and protein structure considerations. Hum. Mutat. *32*, 579–589. https://doi.org/10.1002/humu.21406.
5. de Cima, S., Polo, L.M., Díez-Fernández, C., Martínez, A.I., Cervera, J., Fita, I., and Rubio, V. (2015). Structure of human carbamoyl phosphate synthetase: deciphering the on/off switch of human ureagenesis. Sci. Rep. *5*, 16950. https://doi.org/10.1038/srep16950.
6. Takiguchi, M., and Mori, M. (1995). Transcriptional regulation of genes for ornithine cycle enzymes. Biochem. J. *312*, 649–659. https://doi.org/10.1042/bj3120649.
7. Walker, V. (2014). Ammonia metabolism and hyperammonemic disorders. Adv. Clin. Chem. *67*, 73–150. https://doi.org/10.1016/bs.acc.2014.09.002.
8. Brusilow, S., and Horwich, A. (2001). Urea cycle enzymes, 8th Edition. In The Metabolic Basis of Inherited Disease, C. Scriver, A. Beaudet, D. Valle, W. Sly, B. Childs, K.W. Kinzler, and B. Vogelstein, eds. (McGraw-Hill), pp. 1919–1963.
9. Lichter-Konecki, U. (2016). Defects of the urea cycle. Transl. Sci. Rare Dis. *1*, 23–43. https://doi.org/10.3233/TRD-160002.
10. Diez-Fernandez, C., and Häberle, J. (2017). Targeting CPS1 in the treatment of Carbamoyl phosphate synthetase 1 (CPS1) deficiency, a urea cycle disorder. Expert Opin. Ther. Targets *21*, 391–399. https://doi.org/10.1080/14728222.2017.1294685.
11. Rodney, S., and Boneh, A. (2013). Amino Acid Profiles in Patients with Urea Cycle Disorders at Admission to Hospital due to Metabolic Decompensation. JIMD Rep. *9*, 97–104. https://doi.org/10.1007/8904_2012_186.
12. Haberle, J., Burlina, A., Chakrapani, A., Dixon, M., Karall, D., Lindner, M., Mandel, H., Martinelli, D., Pintos-Morell, G., Santer, R., et al. (2019). Suggested guidelines for the diagnosis and management of urea cycle disorders: First revision. J. Inherit. Metab. Dis. *42*, 1192–1230. https://doi.org/10.1002/jimd.12100.
13. Ah Mew, N., McCarter, R., Daikhin, Y., Lichter-Konecki, U., Nissim, I., Yudkoff, M., and Tuchman, M. (2014). Augmenting ureagenesis in patients with partial carbamyl phosphate synthetase 1 deficiency with N-carbamyl-L-glutamate. J. Pediatr. *165*, 401–403.e3. https://doi.org/10.1016/j.jpeds.2014.04.012.
14. Darwish, A.A., McKiernan, P., and Chardot, C. (2011). Paediatric liver transplantation for metabolic disorders. Part 2: Metabolic disorders with liver lesions. Clin. Res. Hepatol. Gastroenterol. *35*, 271–280. https://doi.org/10.1016/j.clinre.2011.01.008.
15. Darwish, A.A., McKiernan, P., and Chardot, C. (2011). Paediatric liver transplantation for metabolic disorders. Part 1: Liver-based metabolic disorders without liver lesions. Clin. Res. Hepatol. Gastroenterol. *35*, 194–203. https://doi.org/10.1016/j.clinre.2011.01.006.
16. Morioka, D., Kasahara, M., Takada, Y., Shirouzu, Y., Taira, K., Sakamoto, S., Uryuhara, K., Egawa, H., Shimada, H., and Tanaka, K. (2005). Current role of liver transplantation for the treatment of urea cycle disorders: a review of the worldwide English literature and 13 cases at Kyoto University. Liver Transpl. *11*, 1332–1342. https://doi.org/10.1002/lt.20587.
17. Khoja, S., Nitzahn, M., Truong, B., Lambert, J., Willis, B., Allegri, G., Rüfenacht, V., Häberle, J., and Lipshutz, G.S. (2019). A constitutive knockout of murine carbamoyl phosphate synthetase 1 results in death with marked hyperglutaminemia and hyperammonemia. J. Inherit. Metab. Dis. *42*, 1044–1053. https://doi.org/10.1002/jimd.12048.
18. Khoja, S., Nitzahn, M., Hermann, K., Truong, B., Borzone, R., Willis, B., Rudd, M., Palmer, D.J., Ng, P., Brunetti-Pierri, N., and Lipshutz, G.S. (2018). Conditional disruption of hepatic carbamoyl phosphate synthetase 1 in mice results in hyperammonemia without orotic aciduria and can be corrected by liver-directed gene therapy. Mol. Genet. Metab. *124*, 243–253. https://doi.org/10.1016/j.ymgme.2018.04.001.
19. Nitzahn, M., Allegri, G., Khoja, S., Truong, B., Makris, G., Häberle, J., and Lipshutz, G.S. (2020). Split AAV-Mediated Gene Therapy Restores Ureagenesis in a Murine Model of Carbamoyl Phosphate Synthetase 1 Deficiency. Mol. Ther. *28*, 1717–1730. https://doi.org/10.1016/j.ymthe.2020.04.011.
20. Muzyczka, N., and Berns, K.I. (2001). Parvoviridae: The Viruses and Their Replication (Lippincott Williams & Wilkins).
21. Lowes, L.P., Alfano, L.N., Arnold, W.D., Shell, R., Prior, T.W., McColly, M., Lehman, K.J., Church, K., Sproule, D.M., Nagendran, S., et al. (2019). Impact of Age and Motor Function in a Phase 1/2A Study of Infants With SMA Type 1 Receiving Single-Dose Gene Replacement Therapy. Pediatr. Neurol. *98*, 39–45. https://doi.org/10.1016/j.pediatrneurol.2019.05.005.
22. Testa, F., Maguire, A.M., Rossi, S., Pierce, E.A., Melillo, P., Marshall, K., Banfi, S., Surace, E.M., Sun, J., Acerra, C., et al. (2013). Three-year follow-up after unilateral subretinal delivery of adeno-associated virus in patients with Leber congenital Amaurosis type 2. Ophthalmology *120*, 1283–1291. https://doi.org/10.1016/j.ophtha.2012.11.048.
23. George, L.A., Monahan, P.E., Eyster, M.E., Sullivan, S.K., Ragni, M.V., Croteau, S.E., Rasko, J.E.J., Recht, M., Samelson-Jones, B.J., MacDougall, A., et al. (2021). Multiyear Factor VIII Expression after AAV Gene Transfer for Hemophilia A. N. Engl. J. Med. *385*, 1961–1973. https://doi.org/10.1056/NEJMoa2104205.

24. Ramos, J.N., Hollinger, K., Bengtsson, N.E., Allen, J.M., Hauschka, S.D., and Chamberlain, J.S. (2019). Development of Novel Micro-dystrophins with Enhanced Functionality. Mol. Ther. *27*, 623–635. https://doi.org/10.1016/j.ymthe.2019.01.002.

25. Vidovic, D., Carlon, M.S., da Cunha, M.F., Dekkers, J.F., Hollenhorst, M.I., Bijvelds, M.J., Ramalho, A.S., Van den Haute, C., Ferrante, M., Baekelandt, V., et al. (2016). rAAV-CFTRDeltaR Rescues the Cystic Fibrosis Phenotype in Human Intestinal Organoids and Cystic Fibrosis Mice. Am. J. Respir. Crit. Care Med. *193*, 288–298. https://doi.org/10.1164/rccm.201505-0914OC.

26. Toole, J.J., Pittman, D.D., Orr, E.C., Murtha, P., Wasley, L.C., and Kaufman, R.J. (1986). A large region (approximately equal to 95 kDa) of human factor VIII is dispensable for *in vitro* procoagulant activity. Proc. Natl. Acad. Sci. USA *83*, 5939–5942. https://doi.org/10.1073/pnas.83.16.5939.

27. Antunes, M., and Biala, G. (2012). The novel object recognition memory: neurobiology, test procedure, and its modifications. Cogn. Process. *13*, 93–110. https://doi.org/10.1007/s10339-011-0430-z.

28. Shen, W., Le, S., Li, Y., and Hu, F. (2016). SeqKit: A Cross-Platform and Ultrafast Toolkit for FASTA/Q File Manipulation. PLoS One *11*, e0163962. https://doi.org/10.1371/journal.pone.0163962.

29. Tuchman, M., and Yudkoff, M. (1999). Blood levels of ammonia and nitrogen scavenging amino acids in patients with inherited hyperammonemia. Mol. Genet. Metab. *66*, 10–15. https://doi.org/10.1006/mgme.1998.2783.

30. Schnaitman, C., and Greenawalt, J.W. (1968). Enzymatic properties of the inner and outer membranes of rat liver mitochondria. J. Cell Biol. *38*, 158–175. https://doi.org/10.1083/jcb.38.1.158.

31. Raijman, L., and Jones, M.E. (1976). Purification, composition, and some properties of rat liver carbamyl phosphate synthetase (ammonia). Arch. Biochem. Biophys. *175*, 270–278. https://doi.org/10.1016/0003-9861(76)90508-7.

32. Wu, Z., Yang, H., and Colosi, P. (2010). Effect of genome size on AAV vector packaging. Mol. Ther. *18*, 80–86. https://doi.org/10.1038/mt.2009.255.

33. Hermonat, P.L., Quirk, J.G., Bishop, B.M., and Han, L. (1997). The packaging capacity of adeno-associated virus (AAV) and the potential for wild-type-plus AAV gene therapy vectors. FEBS Lett. *407*, 78–84. https://doi.org/10.1016/s0014-5793(97)00311-6.

34. Sarkar, R., Tetreault, R., Gao, G., Wang, L., Bell, P., Chandler, R., Wilson, J.M., and Kazazian, H.H., Jr. (2004). Total correction of hemophilia A mice with canine FVIII using an AAV 8 serotype. Blood *103*, 1253–1260. https://doi.org/10.1182/blood-2003-08-2954.

35. Grieger, J.C., and Samulski, R.J. (2005). Packaging capacity of adeno-associated virus serotypes: impact of larger genomes on infectivity and postentry steps. J. Virol. *79*, 9933–9944. https://doi.org/10.1128/JVI.79.15.9933-9944.2005.

36. Hirsch, M.L., Li, C., Bellon, I., Yin, C., Chavala, S., Pryadkina, M., Richard, I., and Samulski, R.J. (2013). Oversized AAV transductifon is mediated via a DNA-PKcs-independent, Rad51C-dependent repair pathway. Mol. Ther. *21*, 2205–2216. https://doi.org/10.1038/mt.2013.184.

37. Dong, B., Nakai, H., and Xiao, W. (2010). Characterization of genome integrity for oversized recombinant AAV vector. Mol. Ther. *18*, 87–92. https://doi.org/10.1038/mt.2009.258.

38. Scallan, C.D., Lillicrap, D., Jiang, H., Qian, X., Patarroyo-White, S.L., Parker, A.E., Liu, T., Vargas, J., Nagy, D., Powell, S.K., et al. (2003). Sustained phenotypic correction of canine hemophilia A using an adeno-associated viral vector. Blood *102*, 2031–2037. https://doi.org/10.1182/blood-2003-01-0292.

39. Gnatenko, D.V., Saenko, E.L., Jesty, J., Cao, L.X., Hearing, P., and Bahou, W.F. (1999). Human factor VIII can be packaged and functionally expressed in an adeno-associated virus background: applicability to haemophilia A gene therapy. Br. J. Haematol. *104*, 27–36. https://doi.org/10.1046/j.1365-2141.1999.01137.x.

40. Monahan, P.E., Lothrop, C.D., Sun, J., Hirsch, M.L., Kafri, T., Kantor, B., Sarkar, R., Tillson, D.M., Elia, J.R., and Samulski, R.J. (2010). Proteasome inhibitors enhance gene delivery by AAV virus vectors expressing large genomes in hemophilia mouse and dog models: a strategy for broad clinical application. Mol. Ther. *18*, 1907–1916. https://doi.org/10.1038/mt.2010.170.

41. Grose, W.E., Clark, K.R., Griffin, D., Malik, V., Shontz, K.M., Montgomery, C.L., Lewis, S., Brown, R.H., Jr., Janssen, P.M.L., Mendell, J.R., and Rodino-Klapac, L.R. (2012). Homologous recombination mediates functional recovery of dysferlin deficiency following AAV5 gene transfer. PLoS One *7*, e39233. https://doi.org/10.1371/journal.pone.0039233.

42. Haberle, J., Schmidt, E., Pauli, S., Rapp, B., Christensen, E., Wermuth, B., and Koch, H.G. (2003). Gene structure of human carbamylphosphate synthetase 1 and novel mutations in patients with neonatal onset. Hum. Mutat. *21*, 444. https://doi.org/10.1002/humu.9118.

43. Summar, M.L., Hall, L.D., Eeds, A.M., Hutcheson, H.B., Kuo, A.N., Willis, A.S., Rubio, V., Arvin, M.K., Schofield, J.P., and Dawson, E.P. (2003). Characterization of genomic structure and polymorphisms in the human carbamyl phosphate synthetase I gene. Gene *311*, 51–57. https://doi.org/10.1016/s0378-1119(03)00528-6.

44. Nakagawa, T., and Guarente, L. (2009). Urea cycle regulation by mitochondrial sirtuin, SIRT5. Aging *1*, 578–581. https://doi.org/10.18632/aging.100062.

45. Costa, R.H., Grayson, D.R., and Darnell, J.E., Jr. (1989). Multiple hepatocyte-enriched nuclear factors function in the regulation of transthyretin and alpha 1-antitrypsin genes. Mol. Cell Biol. *9*, 1415–1425. https://doi.org/10.1128/mcb.9.4.1415-1425.1989.

46. Wu, Z., Sun, J., Zhang, T., Yin, C., Yin, F., Van Dyke, T., Samulski, R.J., and Monahan, P.E. (2008). Optimization of self-complementary AAV vectors for liver-directed expression results in sustained correction of hemophilia B at low vector dose. Mol. Ther. *16*, 280–289. https://doi.org/10.1038/sj.mt.6300355.

47. Nair, N., Rincon, M.Y., Evens, H., Sarcar, S., Dastidar, S., Samara-Kuko, E., Ghandeharian, O., Man Viecelli, H., Thöny, B., De Bleser, P., et al. (2014). Computationally designed liver-specific transcriptional modules and hyperactive factor IX improve hepatic gene therapy. Blood *123*, 3195–3199. https://doi.org/10.1182/blood-2013-10-534032.

48. Anguela, X., and Elkouby, L. (2022). Optimized Promoter Sequences, Intron-free Expression Constructs and Methods of Use (U.S.P. Office).

49. Levitt, N., Briggs, D., Gil, A., and Proudfoot, N.J. (1989). Definition of an efficient synthetic poly(A) site. Genes Dev. *3*, 1019–1025. https://doi.org/10.1101/gad.3.7.1019.

50. Dong, J.Y., Fan, P.D., and Frizzell, R.A. (1996). Quantitative analysis of the packaging capacity of recombinant adeno-associated virus. Hum. Gene Ther. *7*, 2101–2112. https://doi.org/10.1089/hum.1996.7.17-2101.

51. Haussinger, D., Lamers, W.H., and Moorman, A.F. (1992). Hepatocyte heterogeneity in the metabolism of amino acids and ammonia. Enzyme (Basel) *46*, 72–93. https://doi.org/10.1159/000468779.

52. Dane, A.P., Wowro, S.J., Cunningham, S.C., and Alexander, I.E. (2013). Comparison of gene transfer to the murine liver following intraperitoneal and intraportal delivery of hepatotropic AAV pseudo-serotypes. Gene Ther. *20*, 460–464. https://doi.org/10.1038/gt.2012.67.

53. Chai, S., Wakefield, L., Norgard, M., Li, B., Enicks, D., Marks, D.L., and Grompe, M. (2023). Strong ubiquitous micro-promoters for recombinant adeno-associated viral vectors. Mol. Ther. Methods Clin. Dev. *29*, 504–512. https://doi.org/10.1016/j.omtm.2023.05.013.

54. Lock, M., Alvira, M., Vandenberghe, L.H., Samanta, A., Toelen, J., Debyser, Z., and Wilson, J.M. (2010). Rapid, simple, and versatile manufacturing of recombinant adeno-associated viral vectors at scale. Hum. Gene Ther. *21*, 1259–1271. https://doi.org/10.1089/hum.2010.055.

55. Ewels, P.A., Peltzer, A., Fillinger, S., Patel, H., Alneberg, J., Wilm, A., Garcia, M.U., Di Tommaso, P., and Nahnsen, S. (2020). The nf-core framework for community-curated bioinformatics pipelines. Nat. Biotechnol. *38*, 276–278. https://doi.org/10.1038/s41587-020-0439-x.

56. Ye, X., Robinson, M.B., Pabin, C., Quinn, T., Jawad, A., Wilson, J.M., and Batshaw, M.L. (1997). Adenovirus-mediated *in vivo* gene transfer rapidly protects ornithine transcarbamylase-deficient mice from an ammonium challenge. Pediatr. Res. *41*, 527–534. https://doi.org/10.1203/00006450-199704000-00012.

57. Hatcher, J.P., Jones, D.N., Rogers, D.C., Hatcher, P.D., Reavill, C., Hagan, J.J., and Hunter, A.J. (2001). Development of SHIRPA to characterise the phenotype of gene-targeted mice. Behav. Brain Res. *125*, 43–47. https://doi.org/10.1016/s0166-4328(01)00275-3.

58. Rogers, D.C., Fisher, E.M., Brown, S.D., Peters, J., Hunter, A.J., and Martin, J.E. (1997). Behavioral and functional analysis of mouse phenotype: SHIRPA, a proposed protocol for comprehensive phenotype assessment. Mamm. Genome *8*, 711–713. https://doi.org/10.1007/s003359900551.

59. Lueptow, L.M. (2017). Novel Object Recognition Test for the Investigation of Learning and Memory in Mice. J. Vis. Exp. *126*, 55718. https://doi.org/10.3791/55718.

60. Leger, M., Quiedeville, A., Bouet, V., Haelewyn, B., Boulouard, M., Schumann-Bard, P., and Freret, T. (2013). Object recognition test in mice. Nat. Protoc. *8*, 2531–2537. https://doi.org/10.1038/nprot.2013.155.

61. Kraeuter, A.K., Guest, P.C., and Sarnyai, Z. (2019). The Open Field Test for Measuring Locomotor Activity and Anxiety-Like Behavior. Methods Mol. Biol. *1916*, 99–103. https://doi.org/10.1007/978-1-4939-8994-2_9.

62. Curzon, P., Rustay, N.R., and Browman, K.E. (2009). Cued and Contextual Fear Conditioning for Rodents. In Methods of Behavior Analysis in Neuroscience, 2nd Edition, J.J. Buccafusco, ed. (CRC Press/Taylor & Francis).

63. Cushman, J.D., and Fanselow, M.S. (2010). Fear Conditioning. In Encyclopedia of Behavioral Neuroscience, M.L.M. Koob and R.F. Thompson, eds. (Academic Press), pp. 524–531. https://doi.org/10.1016/B978-0-08-045396-5.00136-6.

64. Matsuda, S., Matsuzawa, D., Ishii, D., Tomizawa, H., and Shimizu, E. (2014). Effects of memory age and interval of fear extinction sessions on contextual fear extinction. Neurosci. Lett. *578*, 139–142. https://doi.org/10.1016/j.neulet.2014.06.054.

OMTN, Volume *36*

Supplemental information

Use of an oversized AAV8 vector for CPS1 deficiency results in long-term survival and ammonia control

Taryn Diep, Wesley Zhou, Rachel E. Reyes, Matthew Nitzahn, Isabel L. Day, Georgios Makris, Lindsay Lueptow, Irina Zhuravka, Stuti Bakshi, Jon Gangoiti, Hyacinth Padaon, Yunfeng Li, Bruce A. Barshop, Johannes Haberle, and Gerald S. Lipshutz

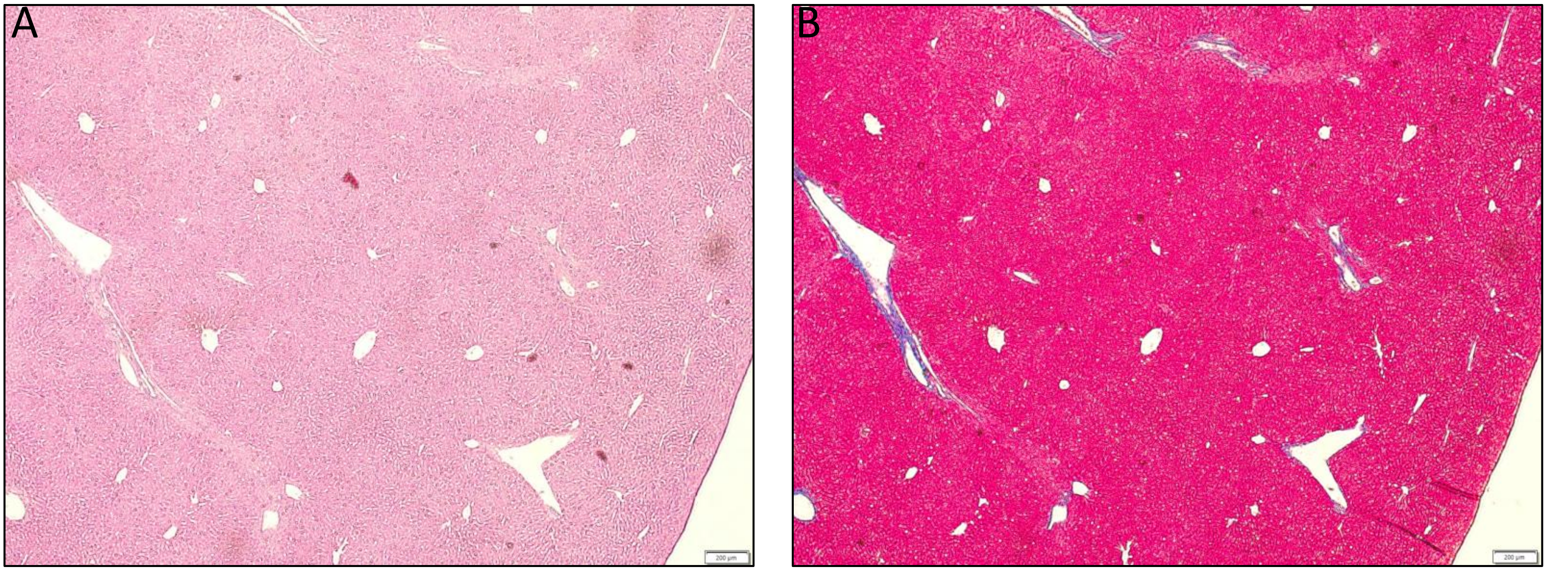

Figure S1. Representative images of age matched Cps1$^{flox/flox}$ liver H&E staining (**A**) demonstrate normal hepatic architecture without lymphocytic infiltrates or evidence of fibrosis on trichrome staining (**B**). Scale bar is 200 μm.

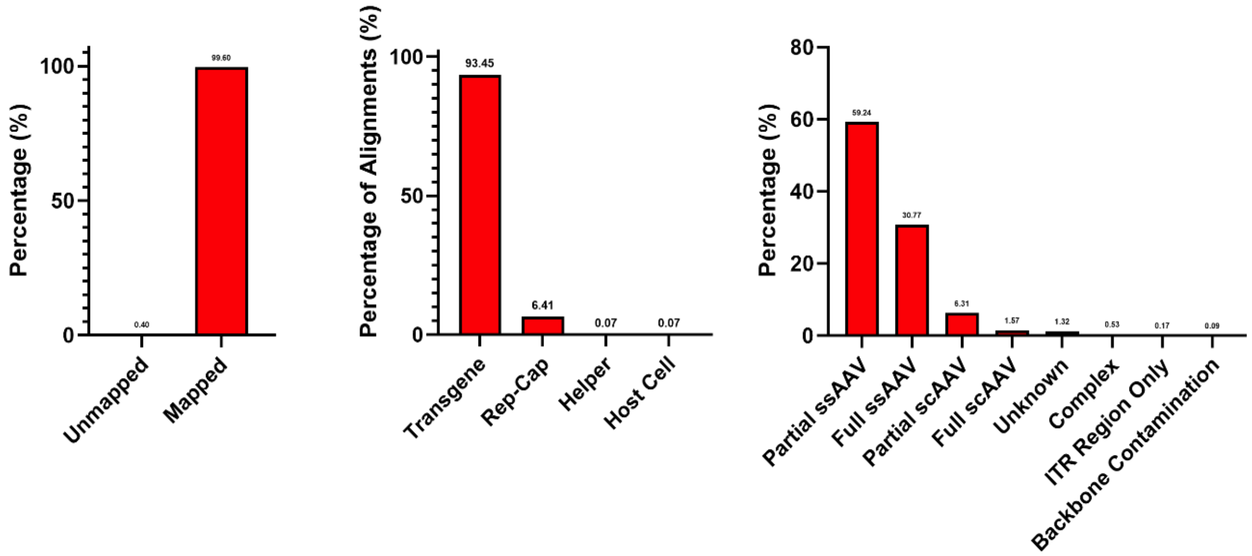

Figure S2. *AAV8.Null Genome Length Fragmentation.* AAV8.Null was barcoded and sequenced using Oxford Nanopore Ligation Sequencing Kit, SQK-LSK114, on a single PromethION™ Flow Cell, and 99.60% of fragments aligned to the created reference sequence (combination of host, helper plasmid, Rep-cap plasmid, and transgene plasmid sequences) **(A)** with a vast majority of alignments (93.45%) mapping to the targeted transgene sequence **(B)**. The percentages of the various AAV transgene genome types indicate the AAV8.Null fragments identified in the sample **(C)**.
